# Compton coincidence in silicon photon-counting CT detectors

**DOI:** 10.1117/1.JMI.9.1.013501

**Published:** 2022-02-08

**Authors:** Christel Sundberg, Mats Danielsson, Mats Persson

**Affiliations:** a KTH Royal Institute of Technology, Physics of Medical Imaging, Stockholm, Sweden; b Prismatic Sensors, part of GE Healthcare, AlbaNova University Center, Stockholm, Sweden; c MedTechLabs, BioClinicum, Karolinska University Hospital, Solna, Sweden

**Keywords:** Compton coincidence, anti-coincidence tracking, tracking detector, silicon detector, computed tomography, photon-counting computed tomography

## Abstract

**Purpose:**

Compton interactions amount to a significant fraction of the registered counts in a silicon detector. In a Compton interaction, only a part of the photon energy is deposited and a single incident photon can result in multiple counts unless tungsten shielding is used. Deep silicon has proved to be a competitive material for photon-counting CT detectors, but to improve the performance further, one possibility is to use coincidence techniques to identify Compton-scattered photons and reconstruct their incident energies.

**Approach:**

In a detector with no tungsten shielding, incident photons can interact through a series of interactions. Based on the position and energy of each interaction, probability-based methods can be used to estimate the incident photon energy. Here, we present a maximum likelihood estimation framework along with an alternative method to estimate the incident photon energy and position in a silicon detector.

**Results:**

Assuming one incident photon per time frame, we show that the incident photon energy can be estimated with a mean error of −0.07±0.03  keV and an RMS error of 3.36±0.02  keV for a realistic case in which we assume a detector with limited energy and spatial resolution. The interaction position was estimated with a mean error of −2±11  μm in x direction and 7±11  μm in y direction. Corresponding RMS errors of 1.09±0.01 and 1.10±0.01  mm were achieved in x and y, respectively.

**Conclusions:**

The presented results show the potential of using probability-based methods to improve the performance of silicon detectors for CT.

## 1 Introduction

Photon-counting spectral detectors are emerging and are predicted to replace scintillators in computed tomography (CT). Contrary to conventional CT using energy-integrating detectors, photon-counting detectors (PCDs) are able to register the energy of each interacting photon. The energy information obtained with a PCD can be used for improved spectral imaging such as energy-weighting or material basis decomposition in which tissue-specific images can be created. Since PCDs are based on semiconductor materials, they are also associated with a lower noise and a higher spatial resolution.^1^^–^^3^

For applications in CT, one research focus has been on cadmium-based detectors: cadmium telluride (CdTe) or cadmium zinc telluride (CZT), whereas another research focus has been on silicon-based detectors, which are currently being evaluated in prototype systems in the clinic. In this work, we will investigate possible further developments on deep silicon detectors. Silicon, in comparison to cadmium-based materials, has a mature manufacturing process and can be manufactured as crystals with a high degree of crystalline perfection. Due to the relatively low attenuation coefficient of silicon, the detector depth in the direction of the incident photons is typically in the order of centimeters. This enables segmentation in which the detector depth can be divided into strata with separate rows of readout channels. The incident photon flux is thereby divided among the segments, which increases the count-rate capability of the detector. Silicon also provides a fast charge collection process due to high charge carrier mobilities and a short distance for the carriers to be collected.

In a silicon detector, each registered event results from either a photoelectric interaction or a Compton interaction. In a photoelectric interaction, the entire photon energy is deposited. For Compton interactions, on the other hand, the photon scatters from an electron and deposits only a fraction of its energy. In a calibrated detector system, Compton interactions provide energy information. However, if the scattered photon interacts again, it is possible to register multiple events from a single incident photon.

**Fig. 1:**
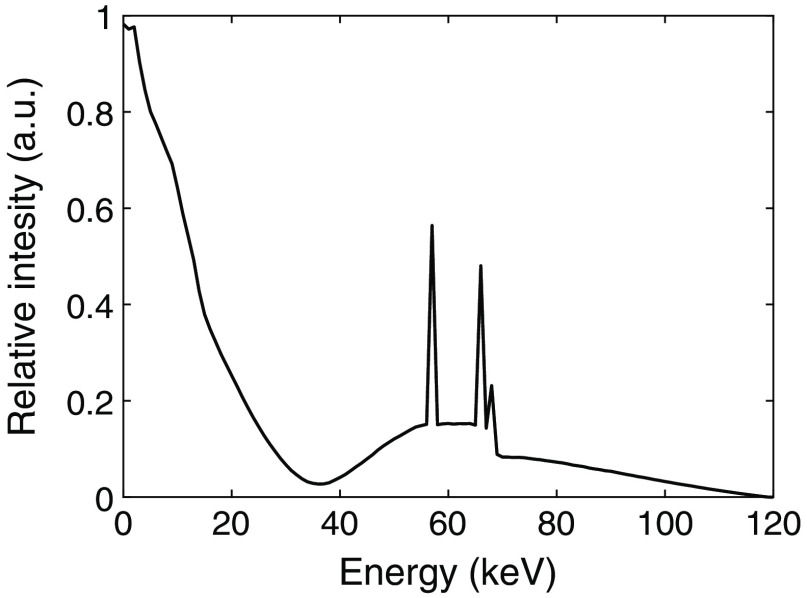
Spectrum of the deposited energies in a silicon detector. The spectrum was simulated based on an x-ray source operated at 120 kVp including 8.48 mm aluminum, 0.8 mm beryllium, and 300 mm soft tissue filtration. For this, a target material of tungsten at an angle of 8 deg was assumed and the IPEM Report 78 on x-ray spectral data was used along with attenuation coefficients from NIST.^5^^,^^6^ The detector response was simulated in GATE based on a previously presented detector geometry of a segmented silicon detector that is 30 mm deep in the direction of the incident photons.^7^^,^^8^

To prevent secondary interactions, tungsten shielding is typically used to absorb scattered photons. The tungsten shield can be aligned with the object scatter collimator so there is no loss on geometrical efficiency. Each registered Compton interaction then corresponds to a unique photon and therefore contributes to dose efficiency and contrast in the image.^4^ Photoelectric and Compton interactions are well-separated with respect to the deposited energy. This can be seen in Fig. 1, which shows a typical spectrum of the deposited energies in a silicon detector with tungsten shielding. The left part of the spectrum, between 0 and 35 keV, shows energies deposited in Compton interactions, and the right part, from 35 to 120 keV, are the resulting photoelectric interactions. Since there is a negligible overlap in deposited energies between the two interaction types, Compton interactions can easily be identified and do not contaminate the photoelectric part of the spectrum. A high energy resolution of the photoelectric interactions can therefore be maintained even in the presence of Compton interactions.

Despite the high performance of silicon detectors with tungsten shielding, it is theoretically possible to achieve detectors with an improved, close to ideal, performance by identifying interactions that belong to the same incident photon. This could be realized using an event-based detector that, instead of counting the number of events at every energy threshold, registers every event with respect to the position, energy, and time of the interaction. The registered information could then be used in combination with the known physics behind Compton scattering to identify interactions. In a detector with no tungsten shielding, secondary interactions can lead to long chains of interactions for single incident photons. If an interaction chain ends in a photoelectric event, the entire photon energy will be deposited within the detector. By identifying all interactions belonging to the same incident photon, the photon energy could be found by adding the deposited energies in the chain of interactions. This introduces the possibility of either reducing the amount of tungsten shielding that is used or removing it entirely to achieve a larger active area and to allow secondary interactions that could improve the detector performance if identified correctly. We have previously presented a method to obtain 1  μm resolution in a silicon detector for CT.^9^ In this work, we propose using a detector with similar spatial resolution to estimate the incident photon energy and position using a maximum likelihood approach based on Compton scattering. We also propose an alternative method in which the incident photon energy is estimated without taking spatial information into account.

Compton cameras that use the dynamics of Compton scattering to infer the direction of incident photons have been presented for many applications including astrophysics and medical imaging.^10^^,^^11^ In medical imaging, Compton cameras have been evaluated for single photon emission computed tomography (SPECT) and also in some cases for positron emission tomography (PET).^12^^,^^13^ In comparison to CT, these imaging techniques are based on incident photons of a single energy with an unknown incident direction. For CT, the case is the opposite: the incident photon direction can be assumed to be known while the photon energy is not. SPECT and PET typically also involve gamma rays while CT uses x-rays. This affects the scattering dynamics since the effect of bound electrons must be taken into account for x-rays. CT is also associated with a high incident photon flux, which increases the number of possible interaction chains and thereby increases the difficulty of correctly identifying interactions from the same incident photon.

This paper describes a first step toward an event-based detector. In this step, we evaluate the performance of estimating the incident photon energy and position under the assumption that the detector is fast enough to resolve one photon at a time. For this, we present a framework for maximum likelihood estimation of the incident photon energy and position in a silicon detector along with an alternative method that estimates the photon energy and position without using Compton scattering dynamics. Based on Monte Carlo simulated photon interactions, we use the developed likelihood framework and the alternative method to estimate the incident photon energy and primary interaction position for a case in which we assume that the interacting photons are well-separated in time. From the estimated energies and interaction positions, we evaluate the performance of the two methods and analyze the resulting likelihood functions. We also provide a discussion of the detector requirements regarding time resolution to implement the presented methods.

## 2 Method

### 2.1 Maximum Likelihood Estimation

Maximum likelihood estimation is a method of estimating the parameters of a statistical model. Given a set of observed data, the goal is to find the parameter values that maximize the likelihood of observing the given data. The likelihood of observing the given data consists of the joint probability distribution of all observed data points based on the assumed statistical model. The resulting parameters that maximize the likelihood function are then the maximum likelihood estimates of the parameters.

In a photon-counting spectral detector, each interacting photon is registered according to the deposited energy and interaction position. An incident photon that interacts in the detector can therefore be described by the number of resulting interactions, N, the position of each interaction $\left\{{\overset{¯}{x}}_{1},\ldots ,{\overset{¯}{x}}_{N}\right\}$ where ${\overset{¯}{x}}_{1}=(x_{1},y_{1},z_{1})$ describes the first interaction and ${\overset{¯}{x}}_{N}=(x_{N},y_{N},z_{N})$ describes the N:th interaction, and the energy deposited in each interaction, $\left\{E_{1},\ldots ,E_{N}\right\}$. In this paper, these entities were used to perform a maximum likelihood estimation of the incident photon energy.

The likelihood function can be calculated as the probability of measuring a certain constellation of interactions given an incident photon of energy $E_{\gamma }$ that enters the detector in position ${\overset{¯}{x}}_{\gamma }=(x_{\gamma },y_{\gamma },z_{\gamma })$. With N, $\left\{{\overset{¯}{x}}_{1},\ldots ,{\overset{¯}{x}}_{N}\right\}$, and $\left\{E_{1},\ldots ,E_{N}\right\}$ as random variables, the likelihood can be expressed as

$P(N=n,\{{\overset{¯}{x}}_{1},\ldots ,{\overset{¯}{x}}_{n}\},\{E_{1},\ldots ,E_{n}\}|E_{\gamma },{\overset{¯}{x}}_{\gamma })$. Given measured data of N, $\left\{{\overset{¯}{x}}_{1},\ldots ,{\overset{¯}{x}}_{N}\right\}$, and $\left\{E_{1},\ldots ,E_{N}\right\}$, a maximum likelihood estimate of $E_{\gamma }$ and ${\bar{x}}_{\gamma }$ is then given by maximizing $P(N=n,\{{\overset{¯}{x}}_{1},\ldots ,{\overset{¯}{x}}_{n}\},\{E_{1},\ldots ,E_{n}\}|E_{\gamma },{\overset{¯}{x}}_{\gamma })$.

The probability of observing a single interaction of energy $E_{1}$ in position ${\bar{x}}_{1}$ given and incident photon in position ${\bar{x}}_{\gamma }$ with energy $E_{\gamma }$, $P(N=1,E_{1},{\overset{¯}{x}}_{1}|E_{\gamma },{\overset{¯}{x}}_{\gamma })$, is based on three probabilities:

$P(\text{interaction}|E_{\gamma },{\overset{¯}{x}}_{\gamma })$: the probability of interacting at all,

$P({\overset{¯}{x}}_{1}|E_{\gamma },{\overset{¯}{x}}_{\gamma })$: the probability of interacting in position ${\overset{¯}{x}}_{1}$ given that the photon interacts,

$P(\text{type}|E_{\gamma },{\overset{¯}{x}}_{\gamma })$: the probability of interaction type (photoelectric or Compton) given that the photon interacts.

**Fig. 2:**
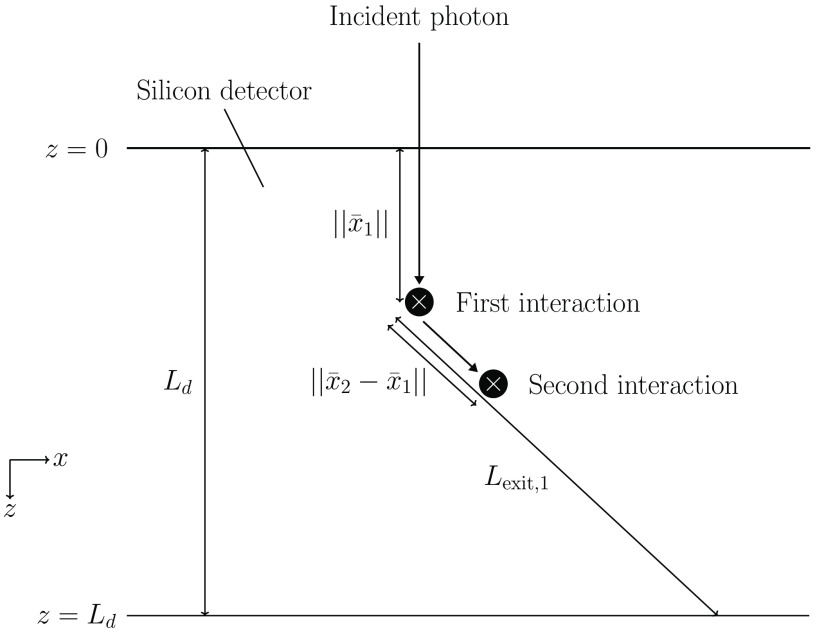
Example of resulting interactions from a single incident photon. The incident photon direction is parallel to the z direction and the detector edges are located at z=0 and $z=L_{d}$. The coordinates of the first interaction are given by ${\overset{¯}{x}}_{1}=(x_{1},y_{1},z_{1})$ and the coordinates of the second interaction are given by ${\overset{¯}{x}}_{2}=(x_{2},y_{2},z_{2})$. For simplicity, $\left(x_{1},y_{1})=(0,0\right)$ was assumed in the figure. The distance $L_{\text{exit},1}$ indicates the distance from the first interaction to the detector edge in the direction of the scattered photon.

The probability of interacting within the distance [0:l] is given by the Beer–Lambert law as $1-e^{-\mu (E_{\gamma })l}$, where e is the base of the natural logarithm and μ is the linear attenuation coefficient of silicon. The probability of an incident photon interacting at all is therefore given as $P(\text{interaction}|E_{\gamma },{\overset{¯}{x}}_{\gamma })=1-e^{-\mu (E_{\gamma })L_{d}}$, where $L_{d}$ is the detector depth in the direction of the incident photon. The probability of a specific interaction position can be obtained by differentiating the Beer-Lambert expression with respect to l. This results in the probability density function $\mu (E_{\gamma })e^{-\mu (E_{\gamma })l}$. Assuming that the incident photon direction is parallel to the z direction and that the detector edges are located at z=0 and $z=L_{d}$, the probability of interacting in position ${\overset{¯}{x}}_{1}$, given that the photon interacts, is obtained by multiplying the probability density function with the length element dx and dividing by the probability of interacting at all: $P({\overset{¯}{x}}_{1}|E_{\gamma },{\overset{¯}{x}}_{\gamma })=\frac{\mu (E_{\gamma })e^{-\mu (E_{\gamma })z_{1}}}{1-e^{-\mu (E_{\gamma })L_{d}}}dx$. It should be noted that having an incident photon direction that is perpendicular to the detector surface is not required. The presented functions can be used for any incident photon direction as long as the direction is known. In Fig. 2, an example of the resulting interactions from an incident photon is shown along with the directions and geometrical references used in this work.

In a silicon detector, each registered interaction is either a photoelectric interaction or a Compton interaction. Rayleigh interactions also occur but cannot be measured since no energy is deposited in the detector. Based on the photon energy range used in CT, pair production does not occur and the effect was therefore not included in the analysis. The probability of each interaction type, $P(\text{Compton}|E_{\gamma },{\overset{¯}{x}}_{\gamma })$ or $P(\text{photo}|E_{\gamma },{\overset{¯}{x}}_{\gamma })$, is obtained by calculating the relative cross sections

$$P(\text{Compton}|E_{\gamma },{\overset{¯}{x}}_{\gamma })=\frac{\sigma {\left(E_{\gamma }\right)}_{\text{Compton}}}{\sigma {\left(E_{\gamma }\right)}_{\text{photo}}+\sigma {\left(E_{\gamma }\right)}_{\text{Compton}}},P(\text{photo}|E_{\gamma },{\overset{¯}{x}}_{\gamma })=\frac{\sigma {\left(E_{\gamma }\right)}_{\text{photo}}}{\sigma {\left(E_{\gamma }\right)}_{\text{photo}}+\sigma {\left(E_{\gamma }\right)}_{\text{Compton}}},$$ (1)

where $\sigma {\left(E_{\gamma }\right)}_{\text{photo}}$ and $\sigma {\left(E_{\gamma }\right)}_{\text{photo}}$ are the photoelectric and Compton cross sections, respectively.

A significant difference between a photoelectric interaction and a Compton interaction is that the entire photon energy is deposited in a photoelectric interaction. In a Compton interaction, on the other hand, a fraction of the photon energy is deposited and a scattered photon either continues to interact in the detector or escapes from the detector. To account for both interaction types, the probability function can be divided into a photoelectric part and a Compton part

$$P(N=1,E_{1},{\overset{¯}{x}}_{1}|E_{\gamma },{\overset{¯}{x}}_{\gamma })=P(N=1,E_{1},{\overset{¯}{x}}_{1},\text{photo}|E_{\gamma },{\overset{¯}{x}}_{\gamma })+P(N=1,E_{1},{\overset{¯}{x}}_{1},\text{Compton}|E_{\gamma },{\overset{¯}{x}}_{\gamma }).$$ (2)

The photoelectric part can be rewritten according to

$$P(N=1,E_{1},{\overset{¯}{x}}_{1},\text{photo}|E_{\gamma },{\overset{¯}{x}}_{\gamma })=P(N=1,E_{1},{\overset{¯}{x}}_{1}|E_{\gamma },{\overset{¯}{x}}_{\gamma },\text{photo})\cdot P(\text{photo}|E_{\gamma },{\overset{¯}{x}}_{\gamma }),$$ (3)

where $P(\text{photo}|E_{\gamma },{\overset{¯}{x}}_{\gamma })$ is the relative cross section from Eq. (1). The remaining function

$P(N=1,{\overset{¯}{x}}_{1},E_{1}|E_{\gamma },{\overset{¯}{x}}_{\gamma },\text{photo})$ consists of the probability of interacting at all, $P(\text{interaction}|E_{\gamma },{\overset{¯}{x}}_{\gamma })$, and the probability of interacting in position ${\overset{¯}{x}}_{1}$, $P({\overset{¯}{x}}_{1}|E_{\gamma },{\overset{¯}{x}}_{\gamma })$. Given a photoelectric interaction, we also expect the entire photon energy to be deposited. In this work, we represent this with a Kronecker delta function δ according to $\delta (E_{1}-E_{\gamma })$. Correspondingly, assuming that the incident photon direction is perpendicular to the detector surface facing the x-ray source, parallel to the z direction, we expect the registered interaction to have the same x and y coordinates as the incident photon: $\left(x_{1},y_{1})=(x_{\gamma },y_{\gamma }\right)$. This can be represented with $\delta ((x_{1},y_{1})-(x_{\gamma },y_{\gamma }))$. Further, assuming detector edges at z=0 and $z=L_{d}$, the total photoelectric part [Eq. (3)] becomes

$$P(N=1,E_{1},{\overset{¯}{x}}_{1},\text{photo}|E_{\gamma },{\overset{¯}{x}}_{\gamma })=\delta ((x_{1},y_{1})-(x_{\gamma },y_{\gamma }))(1-e^{-\mu (E_{\gamma })L_{d}})\cdot \frac{\mu (E_{\gamma })e^{-\mu (E_{\gamma })z_{1}}}{1-e^{-\mu (E_{\gamma })L_{d}}}\delta (E_{1}-E_{\gamma })P(\text{photo}|E_{\gamma },{\overset{¯}{x}}_{\gamma }).$$ (4)

The Compton part can be rewritten analogously to the photoelectric part

$$P(N=1,E_{1},{\overset{¯}{x}}_{1},\text{Compton}|E_{\gamma },{\overset{¯}{x}}_{\gamma })=P(N=1,E_{1},{\overset{¯}{x}}_{1}|E_{\gamma },{\overset{¯}{x}}_{\gamma },\text{Compton})\cdot P(\text{Compton}|E_{\gamma },{\overset{¯}{x}}_{\gamma }),$$ (5)

with $P(\text{Compton}|E_{\gamma },{\overset{¯}{x}}_{\gamma })$ found in Eq. (1). For the Compton case, we introduce two new probabilities: $P(E_{1}|\;E_{\gamma })$, the probability of energy $E_{1}$ being deposited in a Compton interaction, and $P(\text{escape}|E_{\gamma },{\overset{¯}{x}}_{\gamma },E_{1},{\overset{¯}{x}}_{1})$, the probability of the photon escaping the detector without interacting again.

Compton scattering is typically described as a phenomenon in which a photon scatters from a free electron at rest. Given the energy of the incident photon, the energy deposited in the interaction then corresponds to the polar angle of the scattered photon, θ. The probability of depositing a certain energy thereby equals the probability of scattering at the corresponding θ, which can be found using the Klein Nishina formula. However, for the energies used in CT, the effect of bound electrons must be taken into account. This results in a situation where each set of deposited energy $E_{1}$ and incident photon energy $E_{\gamma }$ can occur for multiple values of θ. To account for this, the probability of energy $E_{1}$ being deposited in a Compton interaction is given by

$$P(E_{1}|E_{\gamma })=\int_{0}^{\pi }P(E_{1},\theta |E_{\gamma })d\theta .$$ (6)

The probability of the photon escaping the detector is equal to the probability of the photon not interacting again, which can be calculated using the Beer-Lambert law. This requires the distance to the detector edge in the direction of the scattered photon, $L_{\text{exit},1}$ (see Fig. 2), which can be obtained from the interaction position ${\overset{¯}{x}}_{1}$ and the two scattering angles θ and ϕ. In comparison to the polar scattering angle θ, the azimuthal scattering angle ϕ has a uniform probability distribution and is independent of both the scattered photon energy and θ. Since different combinations of θ and ϕ result in different values of $L_{\text{exit},1}$, the probability of the photon escaping can be calculated as a weighted average based on the probability of ϕ and θ, respectively. This can be written as

$$P(\text{escape}|E_{\gamma },{\overset{¯}{x}}_{\gamma },E_{1},{\overset{¯}{x}}_{1})=\int_{0}^{\pi }\int_{0}^{2\pi }\frac{e^{-\mu (E_{\gamma }-E_{1})L_{\text{exit},1}({\overset{¯}{x}}_{1},\theta ,\phi )}}{2\pi }P(\theta |E_{\gamma },E_{1})d\phi \;d\theta ,$$ (7)

where $P(\theta |E_{\gamma },E_{1})$ is the probability of θ given $E_{1}$ and $E_{\gamma }$. It is worth noting that when the direction of the incident photon is perpendicular to the detector top and bottom surfaces, $L_{\text{exit},1}$ becomes independent of ϕ.

By combining Eqs. (6) and (7) and using the same assumptions as for the photoelectric part, the resulting expression of the Compton part becomes

$$P(N=1,E_{1},{\overset{¯}{x}}_{1},\text{Compton}|E_{\gamma },{\overset{¯}{x}}_{\gamma })=\delta ((x_{1},y_{1})-(x_{\gamma },y_{\gamma }))(1-e^{-\mu (E_{\gamma })L_{d}})\frac{\mu (E_{\gamma })e^{-\mu (E_{\gamma })z_{1}}}{1-e^{-\mu (E_{\gamma })L_{d}}}\int_{0}^{\pi }P(E_{1},\theta |E_{\gamma })d\theta \;\cdot \int_{0}^{\pi }\int_{0}^{2\pi }\frac{e^{-\mu (E_{\gamma }-E_{1})L_{\text{exit},1}({\overset{¯}{x}}_{1},\theta ,\phi )}}{2\pi }P(\theta |E_{\gamma },E_{1})d\phi \;d\theta \cdot P(\text{Compton}|E_{\gamma },{\overset{¯}{x}}_{\gamma }).$$ (8)

The total probability of observing one interaction with energy $E_{1}$ in position ${\overset{¯}{x}}_{1}$ can thereby be written as

$$P(N=1,E_{1},{\overset{¯}{x}}_{1}|E_{\gamma },{\overset{¯}{x}}_{\gamma })=\delta ((x_{1},y_{1})-(x_{\gamma },y_{\gamma }))(1-e^{-\mu (E_{\gamma })L_{d}})\frac{\mu (E_{\gamma })e^{-\mu (E_{\gamma })z_{1}}}{1-e^{-\mu (E_{\gamma })L_{d}}}(\delta (E_{1}-E_{\gamma })P(\text{photo}|E_{\gamma },{\overset{¯}{x}}_{\gamma })\;+\int_{0}^{\pi }P(E_{1},\theta |E_{\gamma })d\theta \int_{0}^{\pi }\int_{0}^{2\pi }\frac{e^{-\mu (E_{\gamma }-E_{1})L_{\text{exit},1}({\overset{¯}{x}}_{1},\theta ,\phi )}}{2\pi }P(\theta |E_{\gamma },E_{1})d\phi \;d\theta \cdot P(\text{Compton}|E_{\gamma },{\overset{¯}{x}}_{\gamma }))$$ (9)

To expand the probability to include more than one interaction, it is important to note that a photoelectric interaction terminates any chain of interactions since the photon is entirely absorbed. Photoelectric events can therefore only be found at the end of an interaction chain and any previous events must be Compton interactions. An interaction chain of N≥2 interactions therefore consists of at least N−1 Compton interactions.

In the case of N=2 interactions, the probability of a first event given by ${\overset{¯}{x}}_{1}$, $E_{1}$ and a second event given by ${\overset{¯}{x}}_{2}$, $E_{2}$ is described as

$$P(N=2,E_{1},E_{2},{\overset{¯}{x}}_{1},{\overset{¯}{x}}_{2}|E_{\gamma },{\overset{¯}{x}}_{\gamma })=\delta ((x_{1},y_{1})-(x_{\gamma },y_{\gamma }))(1-e^{-\mu (E_{\gamma })L_{d}})\frac{\mu (E_{\gamma })e^{-\mu (E_{\gamma })z_{1}}}{1-e^{-\mu (E_{\gamma })L_{d}}}P(\text{Compton}|E_{\gamma },{\overset{¯}{x}}_{\gamma })\;\cdot P(E_{1},\theta_{1}|E_{\gamma })(1-e^{-\mu (E_{\gamma }-E_{1})L_{\text{exit},1}})\frac{\mu (E_{\gamma }-E_{1})e^{-\mu (E_{\gamma }-E_{1})\|{\overset{¯}{x}}_{2}-{\overset{¯}{x}}_{1}\|}}{1-e^{-\mu (E_{\gamma }-E_{1})L_{\mathrm{exit},1}}}\cdot (\delta (E_{2}-(E_{\gamma }-E_{1}))P(\text{photo}|E_{\gamma }-E_{1},{\overset{¯}{x}}_{\gamma })\;+\int_{0}^{\pi }P(E_{2},\theta |E_{\gamma }-E_{1})d\theta \int_{0}^{\pi }\frac{\int_{0}^{2\pi }e^{-\mu (E_{\gamma }-E_{1}-E_{2})L_{\text{exit},2}({\overset{¯}{x}}_{2},\theta ,\phi )}d\phi }{2\pi }P(\theta |E_{\gamma }-E_{1},E_{2})d\theta \;\cdot P(\text{Compton}|E_{\gamma }-E_{1},{\overset{¯}{x}}_{\gamma })).$$ (10)

Correspondingly, the probability of N=n interactions where n>2 is

$$P(N=n,\{E_{1},\ldots ,E_{n}\},\{{\overset{¯}{x}}_{1},\ldots ,{\overset{¯}{x}}_{n}\}|E_{\gamma },{\overset{¯}{x}}_{\gamma })=\delta ((x_{1},y_{1})-(x_{\gamma },y_{\gamma }))\frac{\mu (E_{\gamma })e^{-\mu (E_{\gamma })z_{1}}}{1-e^{-\mu (E_{\gamma })L_{d}}}\;\cdot \overset{n-1}{\underset{k=1}{\prod }}(\frac{\mu (E_{\gamma }-\overset{k}{\underset{i=1}{\sum }}E_{i})e^{-\mu (E_{\gamma }-\overset{k}{\underset{i=1}{\sum }}E_{i})\|{\overset{¯}{x}}_{k+1}-{\overset{¯}{x}}_{k}\|}}{1-e^{-\mu (E_{\gamma }-\overset{k}{\underset{i=1}{\sum }}E_{i})L_{\mathrm{exit},k}}})\cdot P(E_{1},\theta_{1}|E_{\gamma })\overset{n-2}{\underset{k=1}{\prod }}P(E_{k+1},\theta_{k+1}|E_{\gamma }-\overset{k}{\underset{i=1}{\sum }}E_{i})\;\cdot (1-e^{-\mu (E_{\gamma })L_{d}})\overset{n-1}{\underset{k=1}{\prod }}(1-e^{-\mu (E_{\gamma }-\overset{k}{\underset{i=1}{\sum }}E_{i})L_{\text{exit},k}})\cdot P(\text{Compton}|E_{\gamma },{\overset{¯}{x}}_{\gamma })\overset{n-2}{\underset{k=1}{\prod }}P(\text{Compton}|E_{\gamma }-\overset{k}{\underset{i=1}{\sum }}E_{i},{\overset{¯}{x}}_{\gamma })\;\cdot [\delta (E_{n}-(E_{\gamma }-\overset{n-1}{\underset{i=1}{\sum }}E_{i}))\cdot P(\text{photo}|E_{\gamma }-\overset{n-1}{\underset{i=1}{\sum }}E_{i},{\overset{¯}{x}}_{\gamma })+\int_{0}^{\pi }P(E_{n},\theta |E_{\gamma }-\overset{n-1}{\underset{i=1}{\sum }}E_{i})d\theta \cdot \int_{0}^{\pi }\int_{0}^{2\pi }\frac{e^{-\mu (E_{\gamma }-\overset{n}{\underset{i=1}{\sum }}E_{i})L_{\text{exit},n}({\overset{¯}{x}}_{n},\theta ,\phi )}}{2\pi }\cdot P(\theta |E_{\gamma }-\overset{n-1}{\underset{i=1}{\sum }}E_{i},E_{n})d\phi \;d\theta \cdot P(\text{Compton}|E_{\gamma }-\overset{n-1}{\underset{i=1}{\sum }}E_{i},{\overset{¯}{x}}_{\gamma })].$$ (11)

The full derivation of Eqs. (10) and (11) can be found in the Appendix.

#### 2.1.1 Order of interactions

In a typical CT detector we do not expect the temporal resolution to be high enough to resolve the order of the interactions that result from the same incident photon. To estimate $E_{\gamma }$ and ${\overset{¯}{x}}_{\gamma }$ given a set of interactions, the likelihood function must therefore be evaluated for each possible interaction order, e.g., in a case with N=2, with one interaction, interaction a, given by $E_{a}$ and ${\bar{x}}_{a}$, and a second interaction, interaction b, given by $E_{b}$ and ${\bar{x}}_{b}$, the likelihood function becomes

$$P(N=2,E_{a},E_{b},{\overset{¯}{x}}_{a},{\overset{¯}{x}}_{b}|E_{\gamma },{\overset{¯}{x}}_{\gamma })=P(N=2,E_{1}=E_{a},E_{2}=E_{b},{\overset{¯}{x}}_{1}={\overset{¯}{x}}_{a},{\overset{¯}{x}}_{2}={\overset{¯}{x}}_{b}|E_{\gamma },{\overset{¯}{x}}_{\gamma })\;+P(N=2,E_{1}=E_{b},E_{2}=E_{a},{\overset{¯}{x}}_{1}={\overset{¯}{x}}_{b},{\overset{¯}{x}}_{2}={\overset{¯}{x}}_{a}|E_{\gamma },{\overset{¯}{x}}_{\gamma }).$$ (12)

#### 2.1.2 Likelihood function implementation

In this paper, Matlab (The Mathworks inc., Natick, Massachusetts) was used to perform the maximum likelihood estimation. For this, the likelihood function was implemented according to Eqs. (9), (24), and (30) (an example script can be found in the GitHub repository https://github.com/KTH-Physics-of-Medical-Imaging/Compton_coincidence and supplemental data has been uploaded on Zenodo https://zenodo.org/record/5733928#.YeA11NBwF3h). To calculate the constituents of each likelihood function, the linear attenuation coefficient, μ, and the interaction cross sections were obtained from the NIST database.^5^

For Compton interactions, the probability of the deposited energy E and scattering angle θ, $P(E,\theta |E_{\gamma })$, was obtained by simulating photon interactions in GATE^7^ (GEANT4-based simulation platform for emission tomography) using the Penelope model for Compton interactions. The GATE simulation was performed by illuminating a silicon block of size $200\times 200\times 80\;{\mathrm{mm}}^{3}$ with a point source beam perpendicular to the $200\times 200\;{\mathrm{mm}}^{2}$ surface. For each of the energies in the interval [30, 120] keV discretized in steps of 10 keV, 200,000 incident photons were simulated and the resulting interactions were registered with respect to interaction type, position, and deposited energy. Based on the registered interaction type, all primary Compton interactions followed by a Compton or photoelectric event were extracted. From these, the scattering angle was obtained as the angle between the incident photon direction and the line given by the primary interaction and the subsequent Compton or photoelectric interaction. Rayleigh interactions cannot be measured by the detector but are expected to distort the measured scattering angle. To include this effect, interaction chains for which one or many Rayleigh interactions occurred in the time between the primary Compton and the subsequent Compton or photoelectric interaction were also included. The resulting distributions of scattering angles thereby show the effective scattering angles from both Compton and Rayleigh scattering.

**Fig. 3:**
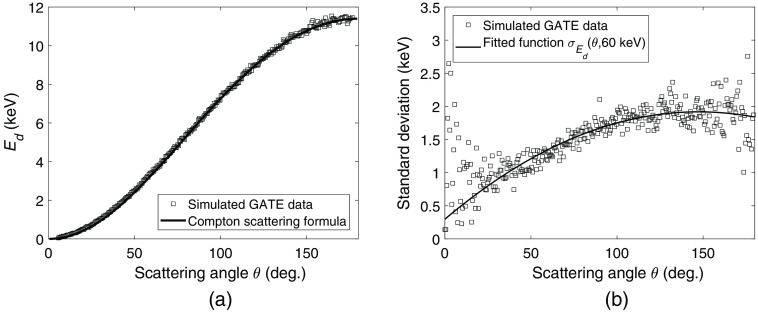
(a) Energy deposited in a Compton interaction at 60 keV as a function of scattering angle. Each simulated GATE value represents the mean deposited energy within a small interval of the scattering angle. (b) Standard deviation of the deposited energy for simulated data along with the fitted curve $\sigma_{E_{d}}$.

For each simulated incident photon energy $E_{\gamma }$ and scattering angle θ, the resulting distribution of deposited photon energies was approximated as a two-term Gaussian function. For both Gaussians, the mean value was set to the deposited energy $E_{d}$ given by the Compton scattering formula

$$\frac{1}{E_{\gamma }-E_{d}}-\frac{1}{E_{\gamma }}=\frac{1}{m_{e}c^{2}}(1-\cos\;\theta ),$$ (13)

where $m_{e}$ is the electron mass and c is the speed of light. A comparison between this approximated mean and the simulated mean can be seen in Fig. 3(a). The standard deviation for each Gaussian was based on the standard deviation of the simulated deposited photon energies, $\sigma_{E_{d}}$. To obtain the relation $\sigma_{E_{d}}(\theta ,E_{\gamma })$, a 2D polynomial was fitted to the surface of $\sigma_{E}$ values obtained at the different scattering angles and simulated incident energies. In Fig. 3(b), the fitted curve $\sigma_{E_{d}}(\theta ,60\;\mathrm{keV})$ is shown along with the standard deviation of the simulated GATE data. Based on the approximated standard deviation, the best fit between the two-term Gaussian function and the simulated data was found for standard deviations $\sigma_{1}=\sigma_{E_{d}}(\theta ,E_{\gamma })$ and $\sigma_{2}=0.18\cdot \sigma_{E_{d}}(\theta ,E_{\gamma })$ resulting in a total function of $\left(\frac{0.5}{\sigma_{1}\sqrt{2\pi }}e^{\frac{-{\left(E-E_{d}\right)}^{2}}{2\sigma_{1}^{2}}}+\frac{0.5}{\sigma_{2}\sqrt{2\pi }}e^{\frac{-{\left(E-E_{d}\right)}^{2}}{2\sigma_{2}^{2}}}\right)$.

Similarly, to obtain the probability of each scattering angle for each simulated incident energy, a histogram of scattering angles was first created for each incident photon energy. A 2D polynomial was then fitted to the histograms resulting in the probability of scattering angle θ given incident photon energy $E_{\gamma }$: $P(\theta |E_{\gamma })$.

The resulting probability of an incident photon of energy $E_{\gamma }$ scattering with angle θ and depositing energy E thereby becomes $P(E,\theta |E_{\gamma })=(\frac{0.5}{\sigma_{1}\sqrt{2\pi }}e^{\frac{-{\left(E-E_{d}\right)}^{2}}{2\sigma_{1}^{2}}}+\frac{0.5}{\sigma_{2}\sqrt{2\pi }}e^{\frac{-{\left(E-E_{d}\right)}^{2}}{2\sigma_{2}^{2}}})\cdot P(\theta |E_{\gamma })$.

Throughout this work, the detector geometry assumed in the likelihood estimations was chosen to achieve a large dose efficiency with respect to the fraction of interacting photons. The detector depth in the direction of the incident photons, parallel to the z direction, $L_{d}$, was therefore set to 80 mm. The area facing the x-ray source was assumed to be unlimited to ensure that interaction chains either end in a photoelectric interaction or a photon escaping the detector. No tungsten shielding was assumed.

The likelihood estimation was implemented for two different cases, first assuming an ideal detector with perfect energy and spatial resolution and then for a more realistic detector with limited energy and spatial resolution. With limited energy and spatial resolution, we expect the observed interactions that act as input to the likelihood estimation to be distorted compared to the ideal case. To account for these potential errors in energy and position, the likelihood estimation was performed using Monte Carlo integration: for each input interaction chain, a large set of possible interaction chains was generated by adding errors in position and energy to the input interaction chain. The likelihood function was then calculated for each of these possible interaction chains and the resulting mean likelihood function was used for the likelihood estimation. Regarding the added errors, errors in the deposited energy were sampled from a Gaussian of zero mean and a standard deviation of $\sigma_{E}=0.5\;\mathrm{keV}$, which corresponds to an estimate of the electronic noise level in a detector with small pixels and therefore a low capacitance. The errors in the registered positions were sampled for each direction x, y, and z based on Gaussian functions with zero mean and standard deviations of $\sigma_{x}=10\;\mu m$, $\sigma_{y}=500\;\mu m$, $\sigma_{z}=500\;\mu m$ in x, y, and z, respectively. Typically, silicon detectors are realized by stacking silicon wafers. However, in this work we make no further assumptions about the detector geometry other than having a large volume of silicon in which we can measure the positions with a certain spatial resolution. One way of realizing this detector could be using a deep silicon detector that is oriented edge-on to the x-ray source. In this work, the assumed spatial resolution corresponds to a narrow strip design presented in our previous work, however, apart from the spatial resolution, no further assumptions were made based on that work.^9^

To improve the robustness of the estimation using Monte Carlo integration, all delta functions with respect to energy in the likelihood expressions were replaced with Gaussian counterparts. These Gaussian functions were chosen as to have a mean corresponding to the total deposited energy and the same standard deviation $\sigma_{E}=0.5\;\mathrm{keV}$ as for the added errors in energy in the Monte Carlo integration.

Due to computational complexity, for each set of interactions, the likelihood function was only optimized with respect to the incident photon energy. Assuming that the maximum of the likelihood function occurs in one of the registered interaction positions, it is only necessary to evaluate the likelihood function in those positions. As seen in Eq. (12), in the case of multiple possible interaction orders, the likelihood function consists of a sum of likelihood functions. However, by only calculating the likelihood function in the interaction positions, it is possible to evaluate and optimize each likelihood term separately. In this paper, for each set of interactions, the incident photon energy and position were estimated according to the likelihood term with the largest maximum. Since each likelihood term corresponds to a specific interaction order, the interaction position was based on the first interaction in the assumed interaction chain.

Throughout the likelihood implementation, energies were discretized in steps of 0.1 keV. This results in a lowest possible interaction energy of 0.1 keV. Likelihood functions involving interactions of 0 keV due to added errors were therefore not evaluated. In a silicon detector, a lowest threshold is typically set to prevent electronic noise from registering as interactions. Any signal below the lowest threshold is therefore ignored, including both noise and interactions in which the deposited energy is below the threshold. A lowest threshold thereby results in the possibility of losing parts of interaction chains below the lowest threshold along with the possibility of registering interaction chains with false interactions resulting from noise. To obtain complete sets of interactions with no missing or extra events, electronic noise was considered only from the perspective of energy resolution in this work and no lowest threshold was therefore included.

### 2.2 Alternative Method of Energy and Position Estimation

To enable a comparison of the performance of the likelihood estimation, an alternative method for energy and position estimation was developed. Since the maximum likelihood method is based on Compton scattering dynamics, the alternative method was designed not to include such features. This was incorporated by estimating the photon energy without including spatial information and, correspondingly, by estimating the incident photon position without considering the deposited energies. Ultimately, this allows for evaluating the effect of including information such as the scattering angle and the distance between interactions when estimating the incident photon energy and position.

For the alternative method, it was first assumed that all registered interactions result from the same incident photon. All interactions below 38.3 keV were then classified as Compton events, remaining interactions were classified as photoelectric events. The threshold of 38.3 keV is based on the Compton scattering formula [Eq. (13)]: given an incident photon of 120 keV, the resulting maximum deposited energy is 38.3 keV. For photons resulting in a single interaction, the position was estimated according to the position of the interaction. In cases with more than one interaction, the position was estimated according to the mean position of the identified Compton interactions. To estimate the energy, in cases with one identified photoelectric interaction, the incident photon energy was estimated as the sum of the deposited energies. In cases with no identified photoelectric interaction, the incident photon energy was estimated based on the maximum of the probability of the incident photon energy $E_{\gamma }$ given the total deposited energy $E_{\mathrm{tot}}$, $P(E_{\gamma }|E_{\mathrm{tot}})$.

To obtain the distribution $P(E_{\gamma }|E_{\mathrm{tot}})$, monoenergetic photons were first simulated in GATE to obtain $P(E|E_{\gamma })$, the probability of deposited energy E given each possible incident photon energy. For this, 10 000 incident photons were simulated for each of the energies in the interval 15 to 120 keV, discretized in steps of 0.1 keV. For all simulations, a detector geometry of $200\times 200\times 80\;{\mathrm{mm}}^{3}$ was used in combination with an illuminated area of $10\times 10\;{\mathrm{mm}}^{2}$. Based on the resulting interactions, the distribution $P(E_{\mathrm{tot}}|E_{\gamma })$ was obtained by adding the deposited energies for all interactions resulting from the same incident photon. To estimate the incident energy based on the total deposited energy, Bayes’ theorem was used to rewrite $P(E_{\mathrm{tot}}|E_{\gamma })$ according to

$$P(E_{\mathrm{tot}}|E_{\gamma })=\frac{P(E_{\gamma }|E_{\mathrm{tot}})P(E_{\mathrm{tot}})}{P(E_{\gamma })}\to P(E_{\gamma }|E_{\mathrm{tot}})=\frac{P(E_{\mathrm{tot}}|E_{\gamma })P(E_{\gamma })}{P(E_{\mathrm{tot}})},$$ (14)

with $P(E_{\gamma })$ as the probability of the incident photon energy and $P(E_{\mathrm{tot}})$ as the probability of the total deposited energy $E_{\mathrm{tot}}$. In this work, $P(E_{\gamma })$ was assumed to be a uniform distribution between 15 and 120 keV.

### 2.3 Monte Carlo Simulation of Photon Interactions

To create input data to the likelihood estimation, a Monte Carlo simulation of photon interactions in a silicon detector was created based on the components of the likelihood function. With $N_{\gamma }$ as the number of incident photons, the Monte Carlo simulation can be described according to Algorithm 1 below.

**Algorithm 1:** Monte Carlo simulation of photon interactions

**for** $k=1:N_{\gamma }$ **do**
Sample incident photon energy $E_{\gamma }$
Sample incident position ${\bar{x}}_{\gamma }$
Sample if the photon interacts or not
**while** photon is interacting **do**
Sample interaction position
Sample interaction type (photoelectric or Compton)
**if** *interaction type is Compton* **then**
Sample scattered photon energy and scattering angles θ and ϕ
Sample if the scattered photon interacts or not
**else if** *interaction type is photoelectric* **then**
Break while loop since no more possible interactions
**end**
**end**
**end**

To sample if the photon interacts or not, where it interacts, and what type of interaction it interacts through (Compton or photoelectric), the previously described $P(\text{interaction}|E_{\gamma },{\overset{¯}{x}}_{\gamma })$, $P({\bar{x}}_{1}|E_{\gamma },{\bar{x}}_{\gamma })$, and $P(\text{type}|E_{\gamma },{\overset{¯}{x}}_{\gamma })$ were used, respectively. In the case of Compton interactions, the probability of scattered photon energy E and scattering angle θ was obtained by sampling from the GATE simulated function $P(E,\theta |\;E_{\gamma })$. For this purpose, the scattering angle θ was discretized in steps of 0.1 radians in the range [0:π]. The scattering angle ϕ was obtained by sampling from a uniform and continuous distribution ranging between 0 and 2π. Throughout the Monte Carlo simulation, an energy discretization of 0.1 keV was used, meaning that the lowest possible interaction energy is 0.1 keV. For all position-dependent probabilities, the linear attenuation coefficient described in Sec. 2.1.2 was used.

### 2.4 60-keV Benchmark

To evaluate the performance of the likelihood estimation, a set of 10,000 incident photons of 60 keV was simulated according to the Monte Carlo simulation described in Sec. 2.3. Although the presented maximum likelihood method is intended for a wide incident spectrum, 60 keV was chosen as a representative energy in CT for simplicity. In the simulations, a detector depth of $L_{d}=80$ mm was assumed in the direction of the incident photons, corresponding to the z direction. The incident photon positions were sampled from an area of $10\times 10\;{\mathrm{mm}}^{2}$ in the xy-plane. Given a photon beam perpendicular to the xy-plane, this means that each primary interaction position will be within the $10\times 10\;{\mathrm{mm}}^{2}$ area. However, for any consecutive interactions, the detector was assumed to be infinite in the x and y directions.

Based on the assumption that each chain of interactions results from a single incident photon, the implemented likelihood framework was used to estimate the incident photon energy and position, first for the ideal detector, assuming perfect energy and spatial resolution in which the simulated interaction chains correspond to the observed interactions, and then for the more realistic detector with a limited energy and spatial resolution as described in Sec. 2.1.2.

For the realistic detector, the observed interaction chains were realized by adding errors in energy and position to the simulated interaction chains. These errors were sampled from the same Gaussian distributions for energy and position as in the Monte Carlo integration described in Sec. 2.1.2. The observed interaction chains were then used as inputs in the energy and position estimation. In the Monte Carlo integration, the number of possible interaction chains was set to 1500 to limit the computational power while maintaining a sufficient representation of both the energy and spatial resolution.

For the alternative method, the incident photon energy and position were estimated for the same simulated interaction chains as for the likelihood method. In the case of the realistic detector, the same realizations of observed interaction chains were used.

To provide a baseline of the performance with the two proposed methods, the energy and position were also estimated for a case in which it was assumed that each interaction corresponds to a unique photon. This corresponds to a typical PCD, for which we expect each registered interaction to correspond to a unique photon, i.e., there is no logic to combine information from multiple interactions. Based on this assumption, the estimated position of the incident photon was set to the position of the interaction. To estimate the incident photon energy, the probability of the incident photon energy $E_{\gamma }$ given the deposited energy E, $P(E_{\gamma }|E)$, was used. This distribution was created in the same way as $P(E_{\gamma }|E_{\mathrm{tot}})$, described in Sec. 2.2, but without adding the deposited energies resulting from the same photon. The energy was estimated according to the maximum of $P(E_{\gamma }|E)$. For this baseline method, the same simulated interaction chains as for the likelihood method and the alternative method were used. It is worth noting that this method results in a larger set of estimated energies and interaction positions compared to the maximum likelihood method and the alternative method since each interaction is assumed to correspond to a single photon.

## 3 Results

### 3.1 Likelihood Function Analysis

**Fig. 4:**
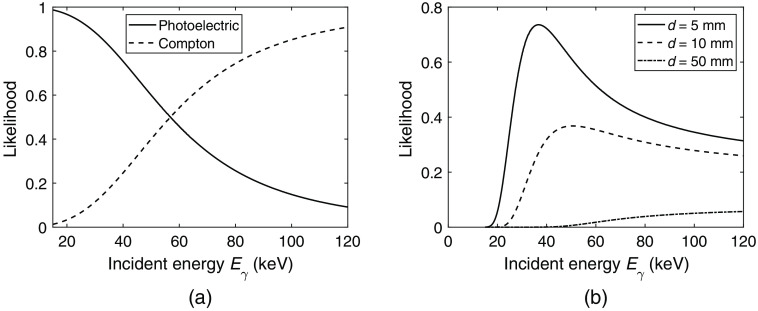
(a) Likelihood of Compton and photoelectric interactions, $P(\text{Compton}|E_{\gamma },{\overset{¯}{x}}_{\gamma })$ and $P(\text{photo}|E_{\gamma },{\overset{¯}{x}}_{\gamma })$, as given by the relative interaction cross sections. (b) Likelihood of interacting after traversed distance d as given by $P(\text{interaction}|E_{\gamma },{\overset{¯}{x}}_{\gamma })P(d|E_{\gamma },{\overset{¯}{x}}_{\gamma })=\mu (E_{\gamma })e^{-\mu (E_{\gamma })d}$ with $E_{\gamma }$ as the incident photon energy.

In Fig. 4(a), the relative interaction cross sections associated with the likelihood of each interaction type, $P(\text{Compton}|E_{\gamma },{\overset{¯}{x}}_{\gamma })$ and $P(\text{photo}|E_{\gamma },{\overset{¯}{x}}_{\gamma })$, are shown as functions of the incident photon energy. Figure 4(b) shows the likelihood of interacting in a specific position based on the traversed distance d. The likelihood is then given by the product $P(\text{interaction}|E_{\gamma },{\overset{¯}{x}}_{\gamma })P(d|E_{\gamma },{\overset{¯}{x}}_{\gamma })=\mu (E_{\gamma })e^{-\mu (E_{\gamma })d}$ with $E_{\gamma }$ as the incident photon energy.

**Fig. 5:**
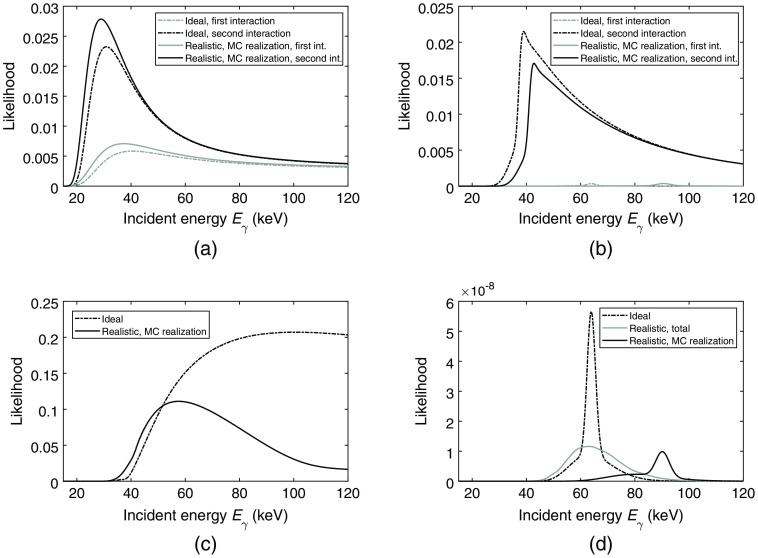
Resulting likelihoods based on the interaction chain in Table 1 assuming the correct order of the interactions. (a)–(d) show the resulting likelihoods for the ideal detector. For the realistic detector, since the total likelihood is calculated through Monte Carlo integration, the likelihood constituents are shown for one Monte Carlo realization. (a) Likelihood according to interaction position. (b) Likelihood according to deposited energy in a Compton interaction. (c) Likelihood of escaping the detector. (d) Total likelihood given as the product of the presented likelihoods in Fig. 4 along with (a)–(c). For the realistic detector, the total likelihood, calculated as the average of 1500 Monte Carlo realizations, is shown along with the resulting likelihood for the single Monte Carlo realization.

**Fig. 6:**
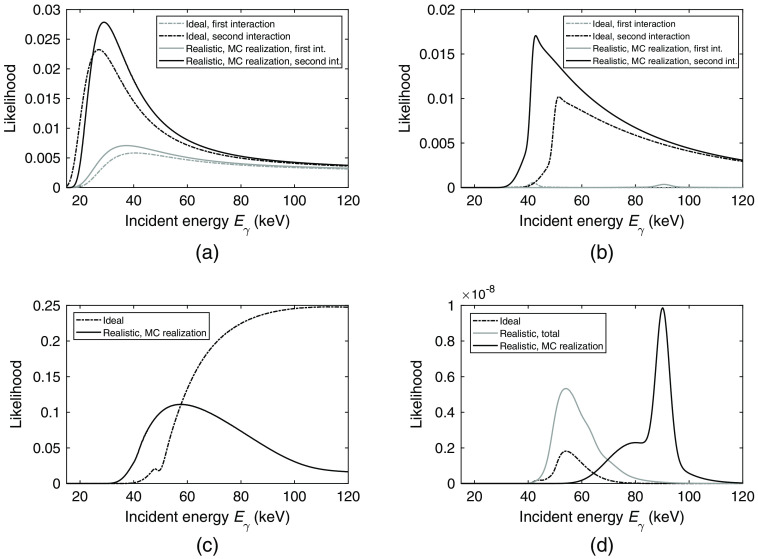
Resulting likelihoods based on the interactions in Table 1 assuming an interaction order in which the first interaction is Interaction 2 and the second interaction is Interaction 1. Through figures (a)–(d), the resulting likelihoods for the ideal detector are shown. For the realistic detector, since the total likelihood is calculated through Monte Carlo integration, the likelihood constituents are shown for one Monte Carlo realization. (a) Likelihood according to interaction position. (b) Likelihood according to deposited energy in a Compton interaction. (c) Likelihood of escaping the detector. (d) Total likelihood given as the product of the presented likelihoods in Fig. 4 along with Figs. 6(a)–6(c). For the realistic detector, the total likelihood, calculated as the average of 1500 Monte Carlo realizations, is shown along with the resulting likelihood for a single Monte Carlo realization based on the likelihoods in Figures (a)–(c).

From the simulated 60-keV photons, an example of an interaction chain consisting of N=2 interactions is given in Table 1. For the given interaction chain, the likelihood functions were calculated for the ideal detector as well as for the realistic detector and the results are shown in Fig. 5. In Fig. 6, the likelihood for the reverse order of the two interactions is shown.

**Table 1:** Example of Monte Carlo simulated interaction chain for N=2 interactions given an incident photon of 60 keV.

Interaction	Deposited energy (keV)	x (mm)	y (mm)	z (mm)	Interaction type
1	7.1	9.670	6.050	6.309	Compton
2	3.2	9.366	4.496	6.310	Compton

### 3.2 Maximum Likelihood Estimation: Performance at 60 keV

The resulting interaction chains from the 10,000 simulated 60-keV photons are presented according to interaction type in Table 2. With 0.33% of the incident photons escaping the detector without interacting at all, a dose efficiency of 99.67% with respect to the fraction of interacting photons is achieved. By including interaction chains of up to four interactions, 97.37% of all interacting photons are included.

**Table 2:** Resulting interaction chains from 10 000 simulated 60-keV photons in a detector that is 80 mm long in the direction of the incident photons.

Interaction chain	Fraction of incident photons (%)
0 interactions	0.33
1 photoelectric	46.07
1 Compton + 1 photoelectric	25.12
2 Compton + 1 photoelectric	12.23
3 Compton + 1 photoelectric	5.63
1 Compton	5.53
2 Compton	1.95
3 Compton	0.40
4 Compton	0.12
Total	97.38

#### 3.2.1 Energy estimation

**Fig. 7:**
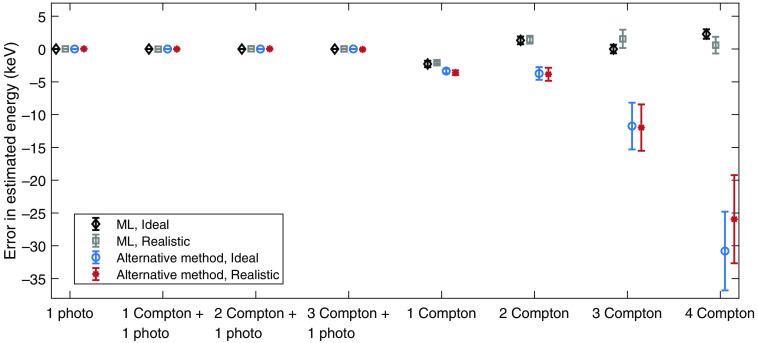
Mean error in estimated energy for each type of interaction chain, estimation method, and detector assumption. For the realistic detector, a limited energy and spatial resolution was assumed. The included error bars indicate the standard error of the mean error in estimated energy.

**Fig. 8:**
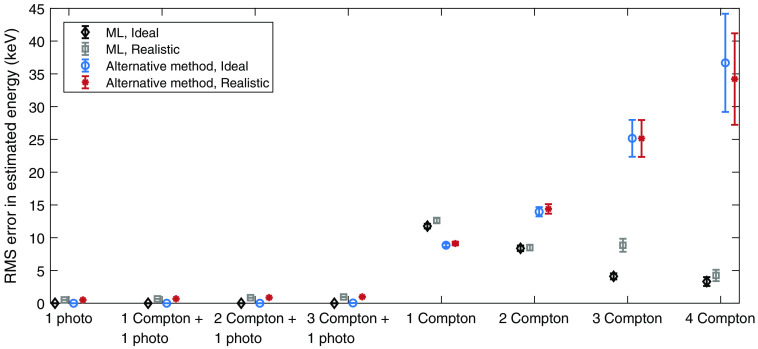
RMS error in estimated energy for each type of interaction chain, estimation method, and detector assumption. For the realistic detector, a limited energy and spatial resolution was assumed. The included error bars indicate the standard error of the RMS error in estimated energy, which was estimated as $1/\sqrt{2n}$ times the RMS error, where n is the number of interaction chains.^15^

The performance with respect to estimating the incident photon energy is shown in Figs. 7 and 8, where Fig. 7 shows the mean error in the energy estimation and Fig. 8 shows the RMS error. For the maximum likelihood estimation, the estimated energies are based on the identified interaction orders, i.e., in cases where the interaction orders were not correctly identified, the estimated mean and RMS errors include estimated energies from incorrectly identified interaction chains.

In Table 3, the total performance considering all analyzed interaction chains is presented with respect to average error and RMS error. Along with the maximum likelihood method and the alternative method, the table also shows the baseline case for which each interaction is assumed to correspond to a unique photon.

**Table 3:** Performance of energy estimation for incident photons of 60 keV. For the alternative method, the energy was estimated based on the probability distribution $P(E_{\gamma }|E_{\mathrm{tot}})$. For the baseline case in which each interaction was assumed to correspond to a unique photon, the energy was estimated based on the probability distribution $P(E_{\gamma }|E)$. For all three cases, a uniform probability distribution between 15 and 120 keV was assumed as the incident spectrum of photon energies. The included intervals represent the standard error of the mean and RMS errors, respectively.

Estimation method	Mean error (keV)		RMS error (keV)	
	Ideal	Realistic	Ideal	Realistic
Maximum likelihood	−0.10±0.03	−0.07±0.03	3.06±0.02	3.36±0.02
Alternative method	−0.35±0.04	−0.36±0.04	3.56±0.03	3.65±0.03
Assuming 1 interaction = 1 photon	−1.23±0.05	−1.28±0.05	6.87±0.04	6.93±0.04

#### 3.2.2 Position estimation

**Fig. 9:**
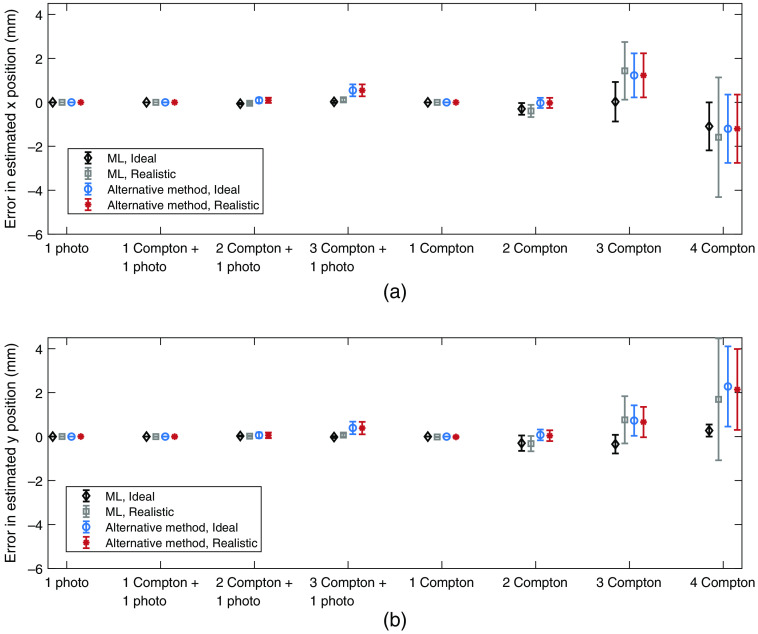
Mean error in the estimated position for each type of interaction chain, estimation method, and detector assumption. For the realistic detector, a limited energy and spatial resolution was assumed. (a) Position estimation in the x direction. (b) Position estimation in the y direction. The included error bars indicate the standard error of the mean error in the estimated position.

**Fig. 10:**
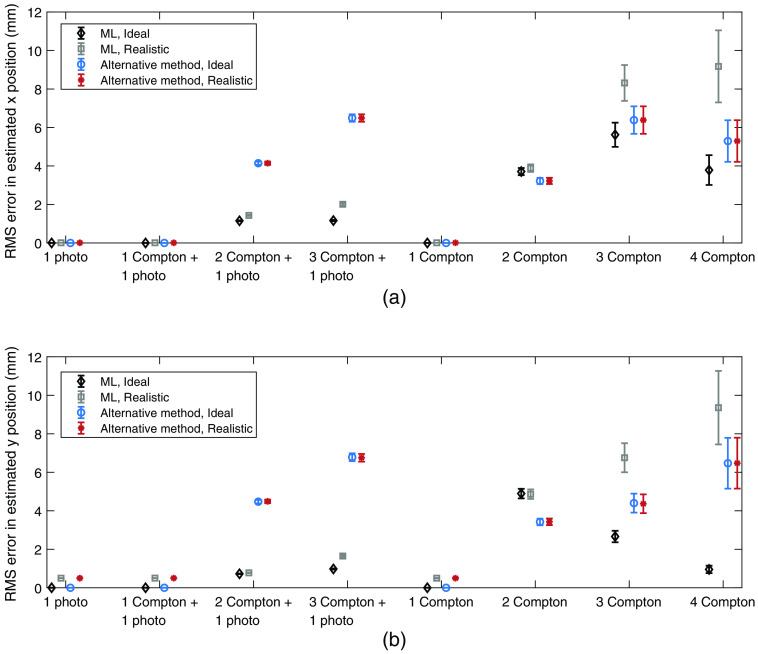
RMS error in the estimated position for each type of interaction chain, estimation method, and detector assumption. For the realistic detector, a limited energy and spatial resolution was assumed. (a) RMS error for the position estimation in the x direction. (b) RMS error for the position estimation in the y direction. The included error bars indicate the standard error of the RMS error in the estimated position, which was estimated as $1/\sqrt{2n}$ times the RMS error, where n is the number of interactions.^15^

For the estimation of the primary interaction position, Figs. 9 and 10 show the mean and RMS errors in the estimated position for each type of interaction chain and direction x and y. Based on all analyzed interaction chains, the resulting mean and RMS errors in the estimated positions are given in Table 4.

**Table 4:** Performance of the position estimation for incident photons of 60 keV. For the alternative method in which the registered interactions were assumed to result for the same incident photon, the position was estimated as the mean position of the identified Compton interactions. In the baseline case for which each interaction was assumed to correspond to a unique incident photon, the position was estimated according to the position of each interaction. The included intervals represent the standard error of the mean and RMS errors, respectively.

	Mean error (mm)			
	x		y	
Estimation method	Ideal	Realistic	Ideal	Realistic
Maximum likelihood	−0.014±0.01	−0.002±0.01	−0.005±0.01	−0.007±0.01
Alternative method	0.05±0.02	0.05±0.02	0.04±0.02	0.04±0.02
1 interaction = 1 photon	0.12±0.05	0.12±0.05	0.10±0.05	0.09±0.05
	RMS error (mm)			
	x		y	
Estimation method	Ideal	Realistic	Ideal	Realistic
Maximum likelihood	0.82±0.01	1.09±0.01	0.80±0.01	1.10±0.01
Alternative method	2.24±0.02	2.24±0.02	2.36±0.02	2.41±0.02
1 interaction = 1 photon	6.54±0.04	6.54±0.04	6.70±0.04	6.72±0.04

With the maximum likelihood method, regarding the interaction order, 98.7% of the analyzed interaction chains were identified according to the correct primary interaction for the ideal case and 97.5% for the realistic detector. For interaction chains of more than one interaction, the corresponding results were 97.2% and 94.6%, respectively. For each interaction type separately, the ratio of correctly identified primary interactions are shown in Table 5.

**Table 5:** Fraction of correctly identified primary interactions using the maximum likelihood method for 10,000 simulated incident photons of 60 keV.

Interaction chain	Correctly identified primary interaction (%)	
	Ideal	Realistic detector
1 photoelectric	100	100
1 Compton + 1 photoelectric	100	100
2 Compton + 1 photoelectric	97.22	92.97
3 Compton + 1 photoelectric	95.91	87.74
1 Compton	100	100
2 Compton	69.74	65.64
3 Compton	77.50	52.50
4 Compton	91.67	75.00

## 4 Discussion

From the relative interaction cross sections shown in Fig. 4, it is clear that photoelectric interactions are most likely between 0 and ∼57  keV. From 57 to 120 keV, Compton interactions are instead the dominating interaction type. Regarding the position-dependent likelihood presented in Fig. 4(b), an incident photon is more likely to interact after a short traversed distance if the photon energy is low. It is seen in the figure that an interaction occurring after a traversed distance of d=5  mm has a clear peak at 37 keV meaning that 37 keV is the most likely incident photon energy given an interaction position that corresponds to a traversed distance of 5 mm. For longer distances, the likelihood is shifted to higher energies. In the curve representing a traversed distance of d=50  mm, the likelihood can be seen to increase with increasing photon energy.

Based on the interaction chain presented in Table 1, the first interaction position corresponds to a traversed distance of 6.309 mm, given by the z coordinate of Interaction 1. This results in a position-dependent likelihood presented in Fig. 5(a), which shows a peak around 40 keV for the first interaction in the ideal case. For the second interaction, the traversed distance between the first and the second interaction is smaller and the resulting position-dependent likelihood for the second interaction therefore shows a sharper peak at a lower incident photon energy. For the Monte Carlo realization, similar curves are shown. The slight shift in position and height of the peaks compared to the ideal case is caused by the included errors that represent limited energy and spatial resolution.

The likelihood of the deposited energy assuming a Compton interaction, shown in Fig. 5(b), shows a narrow peak for the first interaction in both the ideal case and for the Monte Carlo realization that represents the realistic detector. This is due to the scattering angle of the first interaction being known via the two interaction positions. For the Monte Carlo realization, due to the added errors in energy and position, the energy deposited in the first interaction as well as the resulting scattering angle are both lower compared to the ideal case. This shifts the peak of the likelihood to a higher energy. The likelihood of the deposited energy in the second interaction is given by the integral over all possible scattering angles and results in wide curves with sharp peaks to the left. The sharp peaks can be explained via the Compton scattering formula in which the larger the incident photon energy, the larger the possible range of energies for the scattered photon. The likelihood of depositing a certain energy therefore decreases with an increasing photon energy and the highest likelihood is obtained for low photon energies. Due to the effect of bound electrons as well as included Rayleigh interactions, the likelihood curves to the left of the peaks do not tend to zero immediately.

The likelihood of escaping the detector, shown in Fig. 5(c), is dependent on the energy deposited in the last interaction and the direction of the scattered photon. For the presented interaction chain, the scattering angle between Interaction 1 and 2 is ∼π/2  rad, which means that the direction of the interacting photon is parallel to the detector surface. The shortest distance to the detector edge is then obtained if the photon scatters π/2  rad in the second interaction. Since each combination of the incident photon energy and the energy of the scattered photon roughly corresponds to a scattering angle, given the deposited energy of 3.2 keV, the likelihood of escape has its maximum at ∼105  keV for the ideal case. For the realistic detector, due to the added errors that represent energy and spatial resolution, the scattering angle becomes ∼1 radian. The shortest distance to the detector edge is then obtained for a scattering angle that is larger than π/2, which is less likely for all photon energies. Therefore, the resulting likelihood of escape becomes smaller and the corresponding maximum is found slightly below 60 keV.

The total likelihoods in Fig. 5(d) are the product of the likelihoods in Figs. 4 and 5(a)–5(c). Due to the introduced errors in the realistic detector case, there is a widening of the likelihood: the uncertainty in estimating the incident energy has increased. The resulting peaks for the ideal case and the Monte Carlo realization are attributed to the likelihood in Fig. 5(b) in which the likelihood of the first interaction being of certain energy given the scattering angle is very narrow. For the given interaction chain, the likelihood of escape mainly governs the height of the likelihood curves. For the realistic detector, comparing the total likelihood curve based on Monte Carlo integration with the single Monte Carlo realization, the total likelihood curve is more similar to the ideal case and provides a similar estimation of the incident energy.

Regarding the interaction order, Fig. 6 shows the different likelihood constituents based on the interactions in Table 1 in the reverse order. Since both interactions have roughly the same z coordinates, the position-dependent likelihoods for the first interaction, shown in Fig. 6(a) are similar to the corresponding curves for the correct interaction order [Fig. 5(a)]. Given the reverse interaction order, less energy is deposited in the first interaction, which pushes the likelihood curve for the second interaction to the left.

Similar to the case with the correct interaction order, the likelihoods of the deposited energy in Fig. 6(b) appear as peaks for the assumed first interaction. Due to the resulting scattering angles and the difference in deposited energy for the first interaction, the peaks of the likelihood curves are shifted compared to the curves in Fig. 5(b), which assume the correct interaction order. Correspondingly, the likelihood of escape in Fig. 6(c) is based on the reverse order of the interaction positions. Since the scattering angle between the first and second interaction is ∼π/2 regardless of the assumed interaction order, the likelihood curves are similar for both cases. For the ideal case, a local minimum appears at ∼50  keV. For an incident energy of 50 keV, in comparison to the surrounding incident energies, the most likely scattering angle is close to π and therefore results in a lower likelihood of escape. Compared to the case with the correct interaction order, this effect arises due to the higher deposited energy in the assumed second interaction.

The total likelihood of the case with the incorrect interaction order, Fig. 6(d) shows an ideal likelihood function with a maximum at approximately 53 keV. In comparison to the correct interaction order in Fig. 5(d), the maximum of the likelihood function has been shifted to a lower energy. The total likelihood for the realistic detector shows a likelihood curve that is similar to the ideal curve but with a higher amplitude. For the Monte Carlo realization, the resulting total likelihood function has a maximum at ∼90  keV. Since the traversed distance and scattering angle are similar regardless of the interaction order, the appearance of the resulting total likelihood functions is mainly caused by the order in which the energy is deposited. Comparing the ideal case in Figs. 5(b) and 6(b), it is clear that the position of the peak in the likelihood curve for the first interaction changes drastically with interaction order. This peak then largely governs the position of the maximum of the total likelihood function.

For the presented statistics with respect to interaction type in Table 2, the two most common interaction chains, 1 photoelectric and 1 Compton + 1 photoelectric, account for 71% of all registered interactions. It is important to note that the resulting percentages are dependent on the geometry of the detector. In this study, the detector was assumed to be infinite in two directions and 80 mm in the direction parallel to the incident photon direction. Since it is only possible for photons to escape from the detector edges in the z direction, the fraction of long interaction chains is large in comparison to what would be expected if tungsten shielding was used. The detector geometry used in this work provides a case in which the fraction of long interaction chains is close to the maximum. Despite this, it can be seen that most interaction chains consist of few interactions. By including interaction chains of up to four interactions, 97.37% of all interacting photons simulated in this work were included. Based on the presented results, we have shown that the energy and position can be estimated well, especially for short interaction chains. Using an event-based detector, these two features could be combined and thereby enable reconstructing the energies and incident photon positions with good performance. In general, the fact that most interaction chains are short indicates the potential for photon tracking.

In the presented results of the energy and position estimation, the standard error of the mean was calculated according to $\sigma /\sqrt{n}$, with σ as the standard deviation of the mean errors and n as the number of interaction chains. To obtain the standard error of the RMS error, the errors in estimated energies and positions were assumed to be $\chi^{2}$ distributed and the standard error was calculated as $\mathrm{RMS}/\sqrt{2n}$, with RMS as the RMS error.^15^ However, for the 3 Compton and 4 Compton interaction chains, the number of samples is low and the distribution of the errors with respect to both energy and position is non-Gaussian. The standard errors, which build on an assumption of Gaussian distributions, are therefore highly dependent on the specific interactions and should be regarded as approximations.

Regarding the performance of estimating the incident photon energy, shown in Figs. 7 and 8, the energy estimation appears to be most successful for interaction chains that end in photoelectric events, regardless of estimation method. For both estimation methods, the performance also decreases with increasing length of the interaction chain. Regarding the mean error in energy (Fig. 7), the maximum likelihood method and the alternative method show similar performance for interaction chains that involve photoelectric events as well as for the 1 Compton interaction chain. However, for longer interaction chains of only Compton events, the alternative method exhibits worse performance, especially for the 4 Compton interaction chain. Since the alternative method is based on the total deposited energy and does not take the number of interactions into account, this behavior occurs due to the total deposited energy being most likely to result from a single photoelectric event of 15 to 30 keV.

With the maximum likelihood method, the only interaction chain that becomes underestimated with respect to energy is the 1 Compton interaction chain. When the scattering angle is unknown, the likelihood related to Compton scattering has a sharp peak to the left, as can be seen in both Figs. 5(b) and 6(b) for the second (and last) interaction. This causes the maximum of the total likelihood function to be pushed toward the left, resulting in lower estimated energies. For the alternative method, the probability distribution $P(E_{\gamma }|E_{\mathrm{tot}})$ that governs the energy estimation is similar to that of the likelihood related to Compton scattering, which results in a slightly underestimated photon energy as well.

Similar to the mean errors in energy, the RMS errors in the estimated energy shown in Fig. 8 are smaller for interaction chains that end in a photoelectric event. For the realistic detector, in comparison to the ideal detector, the difference in RMS error is mainly caused by the assumed energy resolution of $\sigma_{E}=0.5\;\mathrm{keV}$. With the maximum likelihood method, the highest RMS error occurs for the 1 Compton interaction chain where no scattering angle is known. For the alternative method, the RMS errors are slightly lower for the 1 Compton interaction. However, for longer Compton chains, the RMS errors far exceed the corresponding errors for the maximum likelihood method. Similar to the mean errors, this occurs since the total deposited energy is most probable for a single photoelectric interaction from a low-energy photon.

From Table 3, showing the total performance over all analyzed interactions chains, it is clear that the maximum likelihood method results in the best estimation of the incident photon energy. The presented mean errors indicate that the bias of the maximum likelihood estimation is very small. For the realistic detector, the alternative method exhibits a slightly larger mean error and an RMS error that is approximately 10% higher than for the maximum likelihood method. It is worth noting that for the alternative method, all interaction chains that include an identified photoelectric event are estimated according to the sum of the deposited energies. Apart from the errors that represent energy resolution, the errors in the estimated energies thereby arise from interaction chains with only Compton interactions. Based on Table 2, such interaction chains amount to roughly 8% of all interaction chains. Since the entire photon energy is not deposited in the detector for such interaction chains, the estimated energy will be lower than the actual photon energy, which therefore results in a negative bias. For the maximum likelihood method, the errors in the estimated energies largely also originate from the Compton only interaction chains. However, in the maximum likelihood estimation, interactions are never identified according to interaction type. In the energy estimation, the total likelihood function simultaneously includes the possibility of photoelectric and Compton events.

For the included baseline case in which each interaction is assumed to correspond to a unique photon, the mean error in estimated energy (Table 3) shows that the energy is on average underestimated by 1.23 keV. Correspondingly, for the realistic detector, the RMS error is approximately twice as large as for the maximum likelihood method. Compared to the alternative method, the baseline case also shows significantly worse performance.

Overall, the maximum likelihood method results in the smallest errors with respect to the estimated incident photon energy for both the ideal and the realistic detector. However, since the maximum likelihood functions consist of several probability expressions that must be calculated for each possible incident energy, the computational complexity is much larger compared to the presented alternative method, which is only based on the probability $P(E_{\gamma }|E_{\mathrm{tot}})$. In this work, the simulations were performed on a computer with an Intel Core i9-9980HK CPU (2.40 GHz) and 32 GB RAM. With the maximum likelihood method, the longest computational time per interaction chain was obtained for interaction chains ending in Compton events. With no optimization of the implemented maximum likelihood functions, the computational time was then in the order of minutes, which is mainly due to the evaluation of the probability of escape, which includes integrating overall possible scattering angles [see Eq. (7)]. In comparison, results could be achieved with the alternative method in under a second per interaction chain. However, although the alternative method is more straightforward to implement, we want to emphasize that neither of the two methods has been optimized with respect to computational speed.

In this work, a uniform probability distribution between 15 and 120 keV was assumed regarding the incident photon energies. This was chosen to evaluate the maximum likelihood method without any assumptions regarding the incident photon energies. For future work, it is of interest to include a more realistic spectrum. Since the likelihood functions were derived with no assumptions on the incident spectrum, the assumed spectrum shape can be incorporated by simply multiplying the derived likelihood functions with the probability for each incident photon energy, $E_{\gamma }$, given the spectrum S: $P(E_{\gamma }|S)$. In the case of N=n interactions (where n>2), the total likelihood function then becomes $P(N=n,\{E_{1},\ldots ,E_{n}\},\{{\bar{x}}_{1},\ldots ,{\bar{x}}_{n}\}|E_{\gamma },{\bar{x}}_{\gamma })\cdot P(E_{\gamma }|S)$. However, since we expect the spectrum shape to change depending on projection line, any assumptions on the incident spectrum must be carefully considered.

For the position estimation, the presented mean errors in the estimated positions in Fig. 9 show that the performance decreases with increasing chain length. Similar to the energy estimation, interaction chains that end with photoelectric events show the best performance for both the maximum likelihood method and the alternative method. For the maximum likelihood method, the errors in the estimated positions arise from misplaced Compton interactions: at 60 keV, all resulting photoelectric interactions were correctly identified as being at the end of the interaction chains. An indication of this can be seen in Table 5 for the 1 Compton + 1 photoelectric interaction chain. From Fig. 9 and Table 5, it can also be seen that despite the equal number of Compton interactions in the 2 Compton + 1 photo and the 2 Compton interaction chains, the photoelectric interaction enables a better estimation of the incident photon position. For interaction chains that end in a photoelectric event, the entire photon energy is known, along with all scattering angles. This improves the likelihood estimation of the interaction order. We expect this to also be true for longer interaction chains but due to the low statistics for long chains of Compton events, this cannot be established.

Regarding the RMS errors in the position estimation (Fig. 10), the RMS errors are higher for the alternative method. This is especially clear for the 2 Compton + 1 photo and 3 Compton + 1 photo interaction chains for which the number of interaction chains is high and the statistical uncertainty therefore low. For the y direction [Fig. 10(b)], the RMS errors for the realistic detector are higher compared to the ideal case. This is expected and can be attributed to the assumed energy and spatial resolution. For the maximum likelihood method, there is a clear difference in the RMS errors between the ideal detector and the realistic detector. Since the maximum likelihood estimation includes the angular dependency and the energy deposited along the entire interaction chain, the likelihood of an interaction depends on all previous interactions in the chain. Errors that arise from both the assumed energy and spatial resolution propagate through the interaction chain and can have a large effect on the resulting likelihood function. In comparison, for the alternative method, since the position estimation is based on the mean position of all identified Compton interactions, there is no dependency between the interactions, and the included errors for the realistic detector have a smaller effect on the resulting RMS errors.

Based on the total performance of estimating the incident photon position, given in Table 4, there appears to be no clear difference in performance between the x and y direction, regardless of detector assumption. With the maximum likelihood method, this is mainly due to the fact that it is rare for primary interactions to be misidentified, however, when this happens, the errors in the estimated position can be several centimeters. In the case of the realistic detector with a limited spatial resolution, the errors due to incorrectly identified primary interactions therefore completely mask the effect of the limited spatial resolution. However, for interaction chains with correctly identified primary interactions, the RMS errors are in the same order of magnitude as the assumed spatial resolution. This can be seen in Fig. 10 for the 1 photo, 1 Compton + 1 photo, and 1 Compton interaction chains. Overall, the maximum likelihood method exhibits a larger difference between the ideal and the realistic detector compared to the alternative method and the baseline case. For the realistic detector, the alternative method results in an RMS error that is approximately twice as large as for the maximum likelihood method. For the baseline case in which one interaction is assumed to correspond to a unique incident photon, the position estimation performs worse than the other two methods since the incident interaction position is estimated according to each interaction (photoelectric included). Since many photoelectric interactions are found at the end of interaction chains, the resulting errors in the estimated positions become high. Overall, with both the maximum likelihood method and the alternative method, the results indicate that the position of the incident photon can be determined on a millimeter scale.

In general, the presented results for the maximum likelihood method, given in Tables 3 and 4, exhibit noticeably small errors. This is mainly due to the large fraction of interaction chains that include photoelectric events and or consist of a single interaction. Regarding interaction chains that end in a photoelectric event, as long as the photoelectric interaction is correctly identified, the estimated energy approximately corresponds to the sum of the deposited energies. Correspondingly, it is most likely for the photoelectric interaction to be positioned at the end of the interaction chain because of the high deposited energy. This largely improves the performance of identifying the primary interaction and thereby improves the performance with respect to estimating the incident photon position. For interaction chains in which the primary interaction becomes correctly identified, the resulting errors in the estimated position correspond to the assumed spatial resolution. By combining the interaction statistics in Table 2 with the fraction of correctly identified primary interactions for each interaction type in Table 5, 97.5% of the analyzed interaction chains are identified according to the correct interaction order for the realistic detector. Apart from the errors caused by the assumed spatial resolution, the presented RMS errors in the estimated position mainly occur due to the 2.5% of the incident photons that have misidentified primary interactions. For such photons, the error in the estimated position can be several centimeters.

Further, regarding the energy estimation, we want to emphasize that even for interaction chains consisting of only Compton interactions, the energy can be estimated with an RMS error of ∼11.5  keV using the maximum likelihood method for the realistic detector case. The corresponding bias, given by the mean error in the estimated energy, is then −1.00  keV. For interaction chains consisting of a single Compton interaction, the RMS error is ∼12.6  keV and the mean error is −2.08  keV. Overall, this shows that Compton interactions contribute with valuable energy information.

**Fig. 11:**
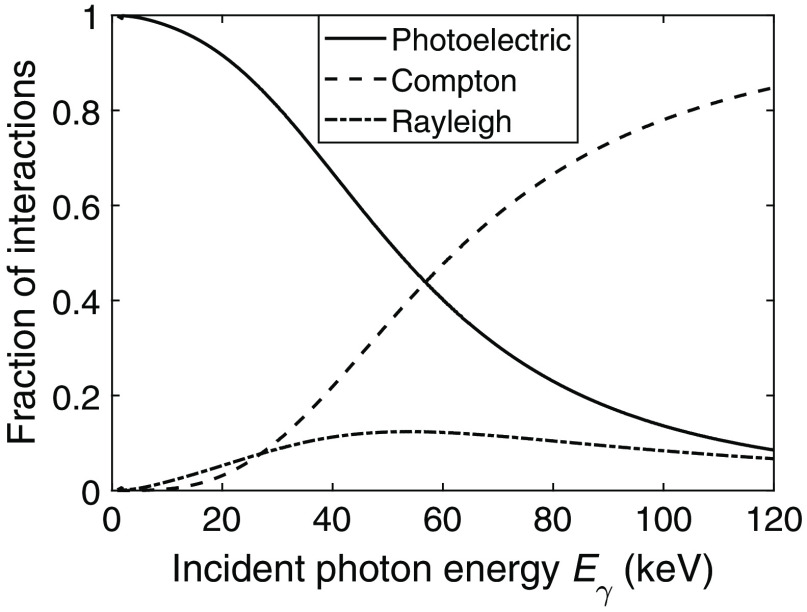
Relative interaction cross section as a function of incident photon energy for photoelectric Compton, and Rayleigh interactions.

For the likelihood estimation in general, apart from the assumed energy and spatial resolution, the effect of Rayleigh interactions decreases the performance of estimating the photon energy and incident position. Since no energy is deposited in a Rayleigh interaction, the detector is unable to track Rayleigh events. However, Rayleigh scattering alters the photon path and distorts the relation between the registered interactions with respect to distance and scattering angle. In this paper, as an approximation, Rayleigh scattering was accounted for in the probability function for Compton scattering $P(E,\theta |\;E_{\gamma })$, which was used both in the Monte Carlo simulation of photon interactions and in the maximum likelihood estimation. For photons between 40 and 60 keV, we expect ∼12% of all interactions to be Rayleigh interactions according to the relative interaction cross sections in Fig. 11.

For future work, it is desirable to evaluate the effect of the lowest threshold. Interactions in which the deposited energy is low typically correspond to a small scattering angle. A lowest threshold that removes low energy events could therefore decrease the estimated energy somewhat but does not necessarily remove the possibility of finding the correct interaction chain. Further, it is also desirable to include the impact of having more than one incident photon. One challenge that arises with several incident photons is that the interactions must be identified correctly to determine the number of incident photons and the incident photon energies. In general, we expect this to become more challenging for interaction chains in which the interactions are far apart. Overall, since all incident photons interact independently of other photons, the likelihood function in Eq. (11) is the building block for the likelihood of any number of incident photons. For example, in the case of two registered interactions, this could possibly result from two photons. Without any assumptions about the x-ray source distribution, we can express these likelihood functions as

$$P(N=2,E_{1},E_{2},{\overset{¯}{x}}_{1},{\overset{¯}{x}}_{2}|E_{\gamma },{\overset{¯}{x}}_{\gamma })\;\text{and}\;P(N=1,E_{1},{\overset{¯}{x}}_{1},|E_{\gamma ,1},{\overset{¯}{x}}_{\gamma ,1})P(N=1,E_{2},{\overset{¯}{x}}_{2},|E_{\gamma ,2},{\overset{¯}{x}}_{\gamma ,2}).$$ (15)

By evaluating each of these likelihoods, it is possible to estimate if the number of incident photons is one or two based on the case that achieves the highest likelihood. Given a set of interactions, it is thereby possible to evaluate the number of incident photons along with the photon energies and incident photon positions by evaluating the likelihood for each combination of possible interaction chains.

To use the presented maximum likelihood method, we expect an event-based detector to be required compared to current detectors for photon-counting CT. Since scattered photons can travel several centimeters before interacting again, interaction chains will likely span over many detector pixels. To find all interactions that belong to the same interaction chain, the search region must therefore be sufficiently large. If we imagine a total search region corresponding to an area of $50\times 50\;{\mathrm{mm}}^{2}$ as seen from the x-ray source and assume a fluence rate of $3.4\cdot 10^{8}\;{\mathrm{mm}}^{-2}\;s^{-1}$, corresponding to the maximum flux in CT for head and intermediate-sized chest images,^16^ the total number of incident photons in the search region becomes $8.5\cdot 10^{11}\;s^{-1}$. To obtain one incident photon per time window, which was the assumption used in this work, the time window must be ∼1  ps. With our presented method, based on the results for a single incident photon per time window, we expect the performance to decrease with an increasing number of incident photons per time window due to the increasing number of interactions. Although this is yet to be evaluated, if the method could handle up to five incident photons during the same time window, the corresponding time window would be ∼6  ps.

Apart from the problem with too many interacting photons during the same time window, pulse pileup can occur if the number of photons per pixel is too large in relation to the speed of the readout electronics. For the presented methods, it should be noted that the problem with pulse pileup will, in itself, be the same as for current CT detectors. Pulse pileup can be mitigated by segmenting the detector in the direction of the incident photons and by reducing the pixel size. However, since the problem of having too many incident photons during the same time window requires a very fast detector, we expect problems with pileup to be smaller compared to in current detectors. Regardless of this, pulse pileup can be included in the likelihood functions by incorporating a pileup model, see e.g., Grönberg et al.^17^ This will mainly result in using flux-dependent spectral response functions that relate the deposited energy to the incident photon energy to include spectral distortion. Further, the assumed count statistics will also be affected since pulse pileup results in non-Poissonian count statistics at high incident fluxes.

Previously proposed detectors include designs both with and without tungsten shielding.^18^ In this work, a detector geometry without tungsten shielding was assumed to allow a large fraction of the incident photons to deposit the entire photon energy. However, we suspect that it might be desirable to include shielding to reduce the size of the required search region by limiting the maximum distance between interactions. On the other hand, this also reduces the interaction chain length, which means that fewer interaction chains will end in photoelectric events. Therefore, there is a trade-off between using tungsten shielding to reduce the number of interactions per detector volume and having interaction chains in which the entire photon energy is deposited in the detector. To evaluate the effect of this on the performance of the presented methods, it is of interest to evaluate the presented methods for various geometries including tungsten shielding. In general, shielding and other geometrical effects, such as gaps between detector elements, can be included in the likelihood functions using position-dependent attenuation coefficients.

If assuming a case in which tungsten shielding is used to create a maximum search region of $25\times 25\;{\mathrm{mm}}^{2}$, the time window corresponding to one and five photons then becomes ∼5 and 24 ps, respectively. Achieving this in current PCDs is associated with many challenges from an engineering point of view. As an example, the time of the interaction is typically not extracted. Instead, whenever the signal is above the lowest threshold, a photon is counted and the interaction energy is sampled every 10 ns during a deadtime period, which is in the order of 100 ns.^19^ However, it has previously been reported that a time resolution of 30 ps can be achieved in a silicon detector.^20^ Based on this, we do not consider this issue to be solved but believe that with engineering ingenuity it might be possible in the near future. This also includes challenges with synchronizing the detector elements. It should also be noted that the image quality is mostly determined by projections with high attenuation in comparison to projections close to the skin-line, which measure the highest fluxes. For a more typical fluence rate in CT of $1\cdot 10^{7}\;{\mathrm{mm}}^{-2}\;s^{-1}$,^2^^,^^16^ the corresponding time windows for one and five photons become 0.16 and 0.8 ns, respectively, which is significantly longer than 30 ps.

## 5 Conclusions

We have presented a maximum likelihood estimation framework along with an alternative method to estimate the incident photon energy in a silicon detector. We have also developed a Monte Carlo simulation of photon interactions. Based on simulated interaction chains, the incident photon energy and position were estimated for 10,000 simulated 60-keV photons assuming a case with one incident photon per time frame. For a non-ideal case with assumed spatial and energy resolution, a mean error in estimated energy of −0.07±0.03  keV was achieved along with an RMS error of 3.36±0.02  keV. With respect to the interaction order, 97.5% of the analyzed interaction chains were identified according to the correct primary interaction using the maximum likelihood method. The interaction position was estimated with a mean error in the order of a few micrometers in both the x and y direction and the corresponding RMS errors were ∼1  mm. Overall, this shows the potential of using event-based detectors. In future work, it is desirable to evaluate the effect of having high incident photon fluxes with multiple incident photons per time window. Further, it is also of interest to explore the effect of using tungsten shielding to reduce the number of coinciding photon interactions.

## 6 Appendix

In the case of N=2 interactions, the probability of a first event given by ${\bar{x}}_{1}$, $E_{1}$ and a second event given by ${\bar{x}}_{2}$, $E_{2}$ can be described as

$$P(N=2,E_{1},E_{2},{\overset{¯}{x}}_{1},{\overset{¯}{x}}_{2}|E_{\gamma },{\overset{¯}{x}}_{\gamma })=P(N=2,E_{1},E_{2},{\overset{¯}{x}}_{1},{\overset{¯}{x}}_{2},1^{\mathrm{st}}\;\text{Compton},2^{\mathrm{nd}}\;\text{photo}|E_{\gamma },{\overset{¯}{x}}_{\gamma })\;+P(N=2,E_{1},E_{2},{\overset{¯}{x}}_{1},{\overset{¯}{x}}_{2},1^{\mathrm{st}}\;\text{Compton},2^{\mathrm{nd}}\;\text{Compton}|E_{\gamma },{\overset{¯}{x}}_{\gamma }).$$ (16)

The first term can then be rewritten according to

$$P(N=2,E_{1},E_{2},{\overset{¯}{x}}_{1},{\overset{¯}{x}}_{2},1^{\mathrm{st}}\;\text{Compton},2^{\mathrm{nd}}\;\text{photo}|E_{\gamma },{\overset{¯}{x}}_{\gamma })=P({\overset{¯}{x}}_{1},1^{\mathrm{st}}\;\text{Compton}|E_{\gamma },{\overset{¯}{x}}_{\gamma })\cdot P(N=2,E_{1},E_{2},{\overset{¯}{x}}_{2},2^{\mathrm{nd}}\;\text{photo}|E_{\gamma },{\overset{¯}{x}}_{\gamma },{\overset{¯}{x}}_{1},1^{\mathrm{st}}\;\text{Compton}),$$ (17)

where

$$P({\overset{¯}{x}}_{1},1^{\mathrm{st}}\;\text{Compton}|E_{\gamma },{\overset{¯}{x}}_{\gamma })=\delta ((x_{1},y_{1})-(x_{\gamma },y_{\gamma }))(1-e^{-\mu (E_{\gamma })L_{d}})\frac{\mu (E_{\gamma })e^{-\mu (E_{\gamma })z_{1}}}{1-e^{-\mu (E_{\gamma })L_{d}}}P(\text{Compton}|E_{\gamma },{\overset{¯}{x}}_{\gamma }),$$ (18)

and

$$P(N=2,E_{1},E_{2},{\overset{¯}{x}}_{2},2^{\mathrm{nd}}\;\text{photo}|E_{\gamma },{\overset{¯}{x}}_{\gamma },{\overset{¯}{x}}_{1},1^{\mathrm{st}}\;\text{Compton})=(1-e^{-\mu (E_{\gamma }-E_{1})L_{\text{exit},1}})\;\cdot \frac{\mu (E_{\gamma }-E_{1})e^{-\mu (E_{\gamma }-E_{1})\|{\overset{¯}{x}}_{2}-{\overset{¯}{x}}_{1}\|}}{1-e^{-\mu (E_{\gamma }-E_{1})L_{\text{exit},1}}}P(E_{1},\theta_{1}|E_{\gamma })\delta (E_{2}-(E_{\gamma }-E_{1}))P(\text{photo}|E_{\gamma }-E_{1},{\overset{¯}{x}}_{\gamma }).$$ (19)

As the scattering angle $\theta_{1}$ can be determined from ${\bar{x}}_{1}$ and ${\bar{x}}_{2}$, the probability of energy $E_{1}$ being deposited can be calculated directly from $P(E_{1},\theta_{1}|E_{\gamma })$ without integrating over θ. The resulting expression of Eq. (17) becomes

$$P(N=2,E_{1},E_{2},{\overset{¯}{x}}_{1},{\overset{¯}{x}}_{2},1^{\mathrm{st}}\;\text{Compton},2^{\mathrm{nd}}\;\text{photo}|E_{\gamma },{\overset{¯}{x}}_{\gamma })=\delta ((x_{1},y_{1})-(x_{\gamma },y_{\gamma }))(1-e^{-\mu (E_{\gamma })L_{d}})\;\cdot \frac{\mu (E_{\gamma })e^{-\mu (E_{\gamma })z_{1}}}{1-e^{-\mu (E_{\gamma })L_{d}}}P(\text{Compton}|E_{\gamma },{\overset{¯}{x}}_{\gamma })(1-e^{-\mu (E_{\gamma }-E_{1})L_{\text{exit},1}})\frac{\mu (E_{\gamma }-E_{1})e^{-\mu (E_{\gamma }-E_{1})\|{\overset{¯}{x}}_{2}-{\overset{¯}{x}}_{1}\|}}{1-e^{-\mu (E_{\gamma }-E_{1})L_{\text{exit},1}}}\;\cdot P(E_{1},\theta_{1}|E_{\gamma })\delta (E_{2}-(E_{\gamma }-E_{1}))P(\text{photo}|E_{\gamma }-E_{1},{\overset{¯}{x}}_{\gamma }).$$ (20)

By rewriting the second term of Eq. (16) we arrive at

$$P(N=2,E_{1},E_{2},{\overset{¯}{x}}_{1},{\overset{¯}{x}}_{2},1^{\mathrm{st}}\;\text{Compton},2^{\mathrm{nd}}\;\text{Compton}|E_{\gamma },{\overset{¯}{x}}_{\gamma })\;=P({\overset{¯}{x}}_{1},1^{\mathrm{st}}\;\text{Compton}|E_{\gamma },{\overset{¯}{x}}_{\gamma })P(N=2,E_{1},E_{2},{\overset{¯}{x}}_{2},2^{\mathrm{nd}}\;\text{Compton}|E_{\gamma },{\overset{¯}{x}}_{\gamma },{\overset{¯}{x}}_{1},1^{\mathrm{st}}\;\text{Compton}),$$ (21)

where the first factor is stated in Eq. (18). The second factor, on the other hand, must take into account a scattered photon as a result of the second Compton interaction. This can be written as

$$P(N=2,E_{1},E_{2},{\overset{¯}{x}}_{2},2^{\mathrm{nd}}\;\text{Compton}|E_{\gamma },{\overset{¯}{x}}_{\gamma },{\overset{¯}{x}}_{1},1^{\mathrm{st}}\;\text{Compton})=P(E_{1},\theta_{1}|E_{\gamma })(1-e^{-\mu (E_{\gamma }-E_{1})L_{\text{exit},1}})\;\cdot \frac{\mu (E_{\gamma }-E_{1})e^{-\mu (E_{\gamma }-E_{1})\|{\overset{¯}{x}}_{2}-{\overset{¯}{x}}_{1}\|}}{1-e^{-\mu (E_{\gamma }-E_{1})L_{\text{exit},1}}}\int_{0}^{\pi }P(E_{2},\theta |E_{\gamma }-E_{1})d\theta \int_{0}^{\pi }\frac{\int_{0}^{2\pi }e^{-\mu (E_{\gamma }-E_{1}-E_{2})L_{\text{exit},2}({\overset{¯}{x}}_{2},\theta ,\phi )}d\phi }{2\pi }\;P(\theta |E_{\gamma }-E_{1},E_{2})d\theta \cdot P(\text{Compton}|E_{\gamma }-E_{1},{\overset{¯}{x}}_{\gamma }),$$ (22)

and results in

$$P(N=2,E_{1},E_{2},{\overset{¯}{x}}_{1},{\overset{¯}{x}}_{2},1^{\mathrm{st}}\;\text{Compton},2^{\mathrm{nd}}\;\text{Compton}|E_{\gamma },{\overset{¯}{x}}_{\gamma })=\delta ((x_{1},y_{1})-(x_{\gamma },y_{\gamma }))(1-e^{-\mu (E_{\gamma })L_{d}})\;\cdot \frac{\mu (E_{\gamma })e^{-\mu (E_{\gamma })z_{1}}}{1-e^{-\mu (E_{\gamma })L_{d}}}P(\text{Compton}|E_{\gamma },{\overset{¯}{x}}_{\gamma })P(E_{1},\theta_{1}|E_{\gamma })(1-e^{-\mu (E_{\gamma }-E_{1})L_{\text{exit},1}})\cdot \frac{\mu (E_{\gamma }-E_{1})e^{-\mu (E_{\gamma }-E_{1})\|{\overset{¯}{x}}_{2}-{\overset{¯}{x}}_{1}\|}}{1-e^{-\mu (E_{\gamma }-E_{1})L_{\text{exit},1}}}\cdot \int_{0}^{\pi }P(E_{2},\theta |E_{\gamma }-E_{1})d\theta \int_{0}^{\pi }\frac{\int_{0}^{2\pi }e^{-\mu (E_{\gamma }-E_{1}-E_{2})L_{\text{exit},2}({\overset{¯}{x}}_{2},\theta ,\phi )}d\phi }{2\pi }P(\theta |E_{\gamma }-E_{1},E_{2})d\theta \cdot P(\text{Compton}|E_{\gamma }-E_{1},{\overset{¯}{x}}_{\gamma }).$$ (23)

By including Eqs. (20) and (23) and identifying the common parts, Eq. (16) can be expressed as

$$P(N=2,E_{1},E_{2},{\overset{¯}{x}}_{1},{\overset{¯}{x}}_{2}|E_{\gamma },{\overset{¯}{x}}_{\gamma })=\delta ((x_{1},y_{1})-(x_{\gamma },y_{\gamma }))(1-e^{-\mu (E_{\gamma })L_{d}})\frac{\mu (E_{\gamma })e^{-\mu (E_{\gamma })z_{1}}}{1-e^{-\mu (E_{\gamma })L_{d}}}P(\text{Compton}|E_{\gamma },{\overset{¯}{x}}_{\gamma })\;\cdot P(E_{1},\theta_{1}|E_{\gamma })(1-e^{-\mu (E_{\gamma }-E_{1})L_{\text{exit},1}})\frac{\mu (E_{\gamma }-E_{1})e^{-\mu (E_{\gamma }-E_{1})\|{\overset{¯}{x}}_{2}-{\overset{¯}{x}}_{1}\|}}{1-e^{-\mu (E_{\gamma }-E_{1})L_{\text{exit},1}}}\cdot (\delta (E_{2}-(E_{\gamma }-E_{1}))P(\text{photo}|E_{\gamma }-E_{1},{\overset{¯}{x}}_{\gamma })\;+\int_{0}^{\pi }P(E_{2},\theta |E_{\gamma }-E_{1})d\theta \int_{0}^{\pi }\frac{\int_{0}^{2\pi }e^{-\mu (E_{\gamma }-E_{1}-E_{2})L_{\text{exit},2}({\overset{¯}{x}}_{2},\theta ,\phi )}d\phi }{2\pi }P(\theta |E_{\gamma }-E_{1},E_{2})d\theta \;\cdot P(\text{Compton}|E_{\gamma }-E_{1},{\overset{¯}{x}}_{\gamma })).$$ (24)

This equation is equal to Eq. (10) in Sec. 2.1.

To generalize this result to N=n interactions (where n>2), we observe that the probability of N=n interactions will consist of two parts: the probability of N Compton interactions and the probability of N−1 Compton interactions followed by a photoelectric interaction. This can be written as

$$P(N=n,\{E_{1},\ldots ,E_{n}\},\{{\overset{¯}{x}}_{1},\ldots ,{\overset{¯}{x}}_{n}\}|E_{\gamma },{\overset{¯}{x}}_{\gamma })\;=P(N=n,\{E_{1},\ldots ,E_{n}\},\{{\overset{¯}{x}}_{1},\ldots ,{\overset{¯}{x}}_{n}\},\{1^{\mathrm{st}}\;\text{Compton},\ldots ,n^{\mathrm{th}}\;\text{Compton}\}|E_{\gamma },{\overset{¯}{x}}_{\gamma })\;+P(N=n,\{E_{1},\ldots ,E_{n}\},\{{\overset{¯}{x}}_{1},\ldots ,{\overset{¯}{x}}_{n}\},\{1^{\mathrm{st}}\;\text{Compton},\ldots ,n-1^{\mathrm{th}}\;\text{Compton}\},n^{\mathrm{th}}\;\text{photo}|E_{\gamma },{\overset{¯}{x}}_{\gamma }).$$ (25)

Similar to Eqs. (17) and (21), the two terms in this expression can each be rewritten. It is important to note that for both the photoelectric part and the Compton part, the first N−1 interactions will be Compton interactions and the probability functions for these interactions will be the same, regardless of whether the last interaction is a Compton or photoelectric event. In Eq. (26), an expansion of the Compton term is shown.

$$P(N=n,\{E_{1},\ldots ,E_{n}\},\{{\overset{¯}{x}}_{1},\ldots ,{\overset{¯}{x}}_{n}\},\{1^{\mathrm{st}}\;\text{Compton},\ldots ,n^{\mathrm{th}}\;\text{Compton}\}|E_{\gamma },{\overset{¯}{x}}_{\gamma })=P({\overset{¯}{x}}_{1},1^{\mathrm{st}}\;\text{Compton}|E_{\gamma },{\overset{¯}{x}}_{\gamma })\cdot P(N=n,\{E_{1},\ldots ,E_{n}\},\{{\overset{¯}{x}}_{2},\ldots ,{\overset{¯}{x}}_{n}\},\{2^{\mathrm{nd}}\;\text{Compton},\ldots ,n^{\mathrm{th}}\mathrm{Compton}\}|E_{\gamma },{\overset{¯}{x}}_{\gamma },{\overset{¯}{x}}_{1},1^{\mathrm{st}}\;\text{Compton})\;=P({\overset{¯}{x}}_{1},1^{\mathrm{st}}\;\text{Compton}|E_{\gamma },{\overset{¯}{x}}_{\gamma })\cdot P(E_{1},{\overset{¯}{x}}_{2},2^{\mathrm{nd}}\;\text{Compton}|E_{\gamma },{\overset{¯}{x}}_{\gamma },{\overset{¯}{x}}_{1},1^{\mathrm{st}}\;\text{Compton})\;\cdot P(N=n,\{E_{2},\ldots ,E_{n}\},\{{\overset{¯}{x}}_{3},\ldots ,{\overset{¯}{x}}_{n}\},\{3^{\mathrm{rd}}\;\text{Compton},\ldots ,n^{\mathrm{th}}\;\text{Compton}\}|E_{\gamma },{\overset{¯}{x}}_{\gamma },E_{1},{\overset{¯}{x}}_{1},{\overset{¯}{x}}_{2},1^{\mathrm{st}}\&2^{\mathrm{nd}}\;\text{Compton})==\ldots =P({\overset{¯}{x}}_{1},1^{\mathrm{st}}\;\text{Compton}|E_{\gamma },{\overset{¯}{x}}_{\gamma })\cdot P(E_{1},{\overset{¯}{x}}_{2},2^{\mathrm{nd}}\;\text{Compton}|E_{\gamma },{\overset{¯}{x}}_{\gamma },{\overset{¯}{x}}_{1},1^{\mathrm{st}}\;\text{Compton})\;\cdot \overset{n-1}{\underset{i=3}{\prod }}P(E_{i-1},{\overset{¯}{x}}_{i},i^{\mathrm{th}}\;\text{Compton}|E_{\gamma },{\overset{¯}{x}}_{\gamma },\{E_{1},\ldots ,E_{i-2}\},\{{\overset{¯}{x}}_{1},\ldots ,{\overset{¯}{x}}_{i-1}\},\{1^{\mathrm{st}}\;\text{Compton},\ldots ,i-1^{\mathrm{th}}\;\text{Compton}\})\cdot P(N=n,E_{n-1},E_{n},{\overset{¯}{x}}_{n},n^{\mathrm{th}}\;\text{Compton}|E_{\gamma },{\overset{¯}{x}}_{\gamma },\{E_{1},\ldots ,E_{n-2}\},\{{\overset{¯}{x}}_{1},\ldots ,{\overset{¯}{x}}_{n-1}\},\{1^{\mathrm{st}}\mathrm{Compton},\ldots ,n-1^{\mathrm{th}}\mathrm{Compton}\}).$$ (26)

It is worth noting that this generalization is applicable for n>3, for n=3, the third factor should be omitted. Looking at the resulting expression, the first factor is found in Eq. (18). The second and third factors are based on identical formulas and can be expressed together as

$$P(E_{1},{\overset{¯}{x}}_{2},2^{\mathrm{nd}}\;\text{Compton}|E_{\gamma },{\overset{¯}{x}}_{\gamma },{\overset{¯}{x}}_{1},1^{\mathrm{st}}\;\text{Compton})\;\cdot \overset{n-1}{\underset{i=3}{\prod }}P(E_{i-1},{\overset{¯}{x}}_{i},i^{\mathrm{th}}\;\text{Compton}|E_{\gamma },{\overset{¯}{x}}_{\gamma },\{E_{1},\ldots ,E_{i-2}\},\{{\overset{¯}{x}}_{1},\ldots ,{\overset{¯}{x}}_{i-1}\},\{1^{\mathrm{st}}\;\text{Compton},\ldots ,i-1^{\mathrm{th}}\;\text{Compton}\})=\overset{n-2}{\underset{k=1}{\prod }}(\frac{\mu (E_{\gamma }-\overset{k}{\underset{i=1}{\sum }}E_{i})e^{-\mu (E_{\gamma }-\overset{k}{\underset{i=1}{\sum }}E_{i})\|{\overset{¯}{x}}_{k+1}-{\overset{¯}{x}}_{k}\|}}{1-e^{-\mu (E_{\gamma }-\overset{k}{\underset{i=1}{\sum }}E_{i})L_{\text{exit},k}}})\cdot P(E_{1},\theta_{1}|E_{\gamma })\overset{n-3}{\underset{k=1}{\prod }}P(E_{k+1},\theta_{k+1}|E_{\gamma }-\overset{k}{\underset{i=1}{\sum }}E_{i})\cdot \overset{n-2}{\underset{k=1}{\prod }}(1-e^{-\mu (E_{\gamma }-\overset{k}{\underset{i=1}{\sum }}E_{i})L_{\text{exit},k}})\cdot \overset{n-2}{\underset{k=1}{\prod }}P(\text{Compton}|E_{\gamma }-\overset{k}{\underset{i=1}{\sum }}E_{i},{\overset{¯}{x}}_{\gamma }).$$ (27)

The fourth and last factor is given as

$$P(N=n,E_{n-1},E_{n},{\overset{¯}{x}}_{n},n^{\mathrm{th}}\;\text{Compton}|E_{\gamma },{\overset{¯}{x}}_{\gamma },\{E_{1},\ldots ,E_{n-2}\},\{{\overset{¯}{x}}_{1},\ldots ,{\overset{¯}{x}}_{n-1}\},\{1^{\mathrm{st}}\;\text{Compton},\ldots ,n-1^{\mathrm{th}}\;\text{Compton}\})=(1-e^{-\mu (E_{\gamma }-\sum_{i=1}^{n-1}E_{i})L_{\text{exit},n-1}})\cdot \;\frac{\mu (E_{\gamma }-\overset{n-1}{\underset{i=1}{\sum }}E_{i})e^{-\mu (E_{\gamma }-\overset{n-1}{\underset{i=1}{\sum }}E_{i})\|{\overset{¯}{x}}_{n}-{\overset{¯}{x}}_{n-1}\|}}{1-e^{-\mu (E_{\gamma }-\overset{n-1}{\underset{i=1}{\sum }}E_{i})L_{\text{exit},n-1}}}\cdot P(E_{n-1},\theta_{n-1}|E_{\gamma }-\overset{n-2}{\underset{i=1}{\sum }}E_{i})\cdot [\int_{0}^{\pi }P(E_{n},\theta |E_{\gamma }-\overset{n-1}{\underset{i=1}{\sum }}E_{i})d\theta \cdot \int_{0}^{\pi }\int_{0}^{2\pi }\frac{e^{-\mu (E_{\gamma }-\overset{n}{\underset{i=1}{\sum }}E_{i})L_{\mathrm{exit},n}({\overset{¯}{x}}_{n},\theta ,\phi )}}{2\pi }\;\cdot P(\theta |E_{\gamma }-\overset{n-1}{\underset{i=1}{\sum }}E_{i},E_{n})d\phi \;d\theta \cdot P(\text{Compton}|E_{\gamma }-\overset{n-1}{\underset{i=1}{\sum }}E_{i},{\overset{¯}{x}}_{\gamma })].$$ (28)

Regarding the corresponding expression for the photoelectric part, since the probability functions of the first N−1 interactions are the same as for the Compton case, the only difference is the probability of the last interaction [factor four in Eq. (26)]. For the photoelectric case, this factor instead becomes

$$P(N=n,E_{n-1},E_{n},{\overset{¯}{x}}_{n},n^{\mathrm{th}}\;\text{photo}|E_{\gamma },{\overset{¯}{x}}_{\gamma },\{E_{1},\ldots ,E_{n-2}\},\{{\overset{¯}{x}}_{1},\ldots ,{\overset{¯}{x}}_{n-1}\},\{1^{\mathrm{st}}\;\text{Compton},\ldots ,n-1^{\mathrm{th}}\;\text{Compton}\})\;=(1-e^{-\mu (E_{\gamma }-\sum_{i=1}^{n-1}E_{i})L_{\text{exit},n-1}})\frac{\mu (E_{\gamma }-\overset{n-1}{\underset{i=1}{\sum }}E_{i})e^{-\mu (E_{\gamma }-\overset{n-1}{\underset{i=1}{\sum }}E_{i})\|{\overset{¯}{x}}_{n}-{\overset{¯}{x}}_{n-1}\|}}{1-e^{-\mu (E_{\gamma }-\overset{n-1}{\underset{i=1}{\sum }}E_{i})L_{\text{exit},n-1}}}\;\cdot P(E_{n-1},\theta_{n-1}|E_{\gamma }-\overset{n-2}{\underset{i=1}{\sum }}E_{i})\cdot [\delta (E_{n}-(E_{\gamma }-\overset{n-1}{\underset{i=1}{\sum }}E_{i}))\cdot P(\text{photo}|E_{\gamma }-\overset{n-1}{\underset{i=1}{\sum }}E_{i},{\overset{¯}{x}}_{\gamma })].$$ (29)

Finally, by combining Eqs. (26)–(29) via Eq. (25), the full expression for the probability of N=n interactions becomes

$$P(N=n,\{E_{1},\ldots ,E_{n}\},\{{\overset{¯}{x}}_{1},\ldots ,{\overset{¯}{x}}_{n}\}|E_{\gamma },{\overset{¯}{x}}_{\gamma })=\delta ((x_{1},y_{1})-(x_{\gamma },y_{\gamma }))\frac{\mu (E_{\gamma })e^{-\mu (E_{\gamma })z_{1}}}{1-e^{-\mu (E_{\gamma })L_{d}}}\;\cdot \overset{n-1}{\underset{k=1}{\prod }}(\frac{\mu (E_{\gamma }-\overset{k}{\underset{i=1}{\sum }}E_{i})e^{-\mu (E_{\gamma }-\overset{k}{\underset{i=1}{\sum }}E_{i})\|{\overset{¯}{x}}_{k+1}-{\overset{¯}{x}}_{k}\|}}{1-e^{-\mu (E_{\gamma }-\overset{k}{\underset{i=1}{\sum }}E_{i})L_{\mathrm{exit},k}}})\cdot P(E_{1},\theta_{1}|E_{\gamma })\overset{n-2}{\underset{k=1}{\prod }}P(E_{k+1},\theta_{k+1}|E_{\gamma }-\overset{k}{\underset{i=1}{\sum }}E_{i})\;\cdot (1-e^{-\mu (E_{\gamma })L_{d}})\overset{n-1}{\underset{k=1}{\prod }}(1-e^{-\mu (E_{\gamma }-\overset{k}{\underset{i=1}{\sum }}E_{i})L_{\text{exit},k}})\cdot P(\text{Compton}|E_{\gamma },{\overset{¯}{x}}_{\gamma })\overset{n-2}{\underset{k=1}{\prod }}P(\text{Compton}|E_{\gamma }-\overset{k}{\underset{i=1}{\sum }}E_{i},{\overset{¯}{x}}_{\gamma })\;\cdot [\delta (E_{n}-(E_{\gamma }-\overset{n-1}{\underset{i=1}{\sum }}E_{i}))\cdot P(\text{photo}|E_{\gamma }-\overset{n-1}{\underset{i=1}{\sum }}E_{i},{\overset{¯}{x}}_{\gamma })+\int_{0}^{\pi }P(E_{n},\theta |E_{\gamma }-\overset{n-1}{\underset{i=1}{\sum }}E_{i})d\theta \;\cdot \int_{0}^{\pi }\int_{0}^{2\pi }\frac{e^{-\mu (E_{\gamma }-\overset{n}{\underset{i=1}{\sum }}E_{i})L_{\text{exit},n}({\overset{¯}{x}}_{n},\theta ,\phi )}}{2\pi }P(\theta |E_{\gamma }-\overset{n-1}{\underset{i=1}{\sum }}E_{i},E_{n})d\phi \;d\theta \cdot P(\mathrm{Compton}|E_{\gamma }-\overset{n-1}{\underset{i=1}{\sum }}E_{i},{\overset{¯}{x}}_{\gamma })].$$ (30)

This equation corresponds to Eq. (11) in Sec. 2.1.
